# Novel mechanisms to inhibit HIV reservoir seeding using Jak inhibitors

**DOI:** 10.1371/journal.ppat.1006740

**Published:** 2017-12-21

**Authors:** Christina Gavegnano, Jessica H. Brehm, Franck P. Dupuy, Aarthi Talla, Susan Pereira Ribeiro, Deanna A. Kulpa, Cheryl Cameron, Stephanie Santos, Selwyn J. Hurwitz, Vincent C. Marconi, Jean-Pierre Routy, Laurent Sabbagh, Raymond F. Schinazi, Rafick Pierre Sékaly

**Affiliations:** 1 Center for AIDS Research, Laboratory of Biochemical Pharmacology, Department of Pediatrics, Emory University, Atlanta, GA, United States of America; 2 Case Western Reserve University, Dept. of Pathology, Cleveland, OH, United States of America; 3 Research Institute of the MUHC, Montréal, QC, Canada; 4 Unconditional Love, Melbourne, FL, United States of America; 5 Division of Infectious Diseases, Emory University School of Medicine, Atlanta, GA, United States of America; 6 Chronic Viral Illnesses Service Research Institute, Division of Hematology, McGill University Health Centre, Montréal, QC, Canada; 7 Université de Montréal, Department of Microbiology, Infectiology, and Immunology, Montreal, QC, Canada; Vaccine Research Center, UNITED STATES

## Abstract

Despite advances in the treatment of HIV infection with ART, elucidating strategies to overcome HIV persistence, including blockade of viral reservoir establishment, maintenance, and expansion, remains a challenge. T cell homeostasis is a major driver of HIV persistence. Cytokines involved in regulating homeostasis of memory T cells, the major hub of the HIV reservoir, trigger the Jak-STAT pathway. We evaluated the ability of tofacitinib and ruxolitinib, two FDA-approved Jak inhibitors, to block seeding and maintenance of the HIV reservoir *in vitro*. We provide direct demonstration for involvement of the Jak-STAT pathway in HIV persistence *in vivo*, *ex vivo*, and *in vitro*; pSTAT5 strongly correlates with increased levels of integrated viral DNA *in vivo*, and *in vitro* Jak inhibitors reduce the frequency of CD4^+^ T cells harboring integrated HIV DNA. We show that Jak inhibitors block viral production from infected cells, inhibit γ-C receptor cytokine (IL-15)-induced viral reactivation from latent stores thereby preventing transmission of infectious particles to bystander activated T cells. These results show that dysregulation of the Jak-STAT pathway is associated with viral persistence *in vivo*, and that Jak inhibitors target key events downstream of γ-C cytokine (IL-2, IL-7 and IL-15) ligation to their receptors, impacting the magnitude of the HIV reservoir in all memory CD4 T cell subsets *in vitro* and *ex vivo*. Jak inhibitors represent a therapeutic modality to prevent key events of T cell activation that regulate HIV persistence and together, specific, potent blockade of these events may be integrated to future curative strategies.

## Author summary

HIV persists in infected hosts in a small number of CD4 + memory T cells as latently infected proviruses. Homeostatic cytokines, which play a major role in the maintenance of T cell memory, also enable the persistence of latently infected cells. The pathways downstream of these homeostatic cytokines are well known and drugs that target these pathways have been developed and have been safely used in inflammatory diseases and in myelofibrosis. We have used these drugs to inhibit the maintenance and the spread of HIV infected cells carrying latent forms of the virus. We show that ruxolitinib and tofacitinib will inhibit the expansion of these cells and their capacity to infect other cells upon reactivation. This class of drugs is currently being tested in clinical trials.

## Introduction

Current antiretroviral therapy (ART) has yielded significant success in achieving long-term suppression of viral load and in improving survival of HIV infected subjects [1–4]. Even so, ART fails to eliminate a small number of cells harboring integrated, replication competent viral DNA. This HIV reservoir has represented a major limitation in eradicating HIV. The HIV reservoir has been shown to persist in central memory T_CM_, transitional memory T_TM_ and effector memory T_EM_ CD4 T cells which require exposure to γ-C receptor cytokines for their long-term persistence [5, 6]. IL-2, IL-7 and IL-15 are γ-C receptor, homeostatic cytokines involved in the maintenance of T cell memory and which activate STAT5 mediated signaling [7]. In addition, the Jak-STAT pathway is also triggered by type I and type II Interferons, two important mediators of inflammation in viral infections including HIV [8–11]. Initial attempts to purge HIV involved the use of IL-2; results of these studies while promising, since virus was undetectable, did not reach their objective as viral load rebounded upon cessation of therapy [12]. We have previously shown that IL-7 driven homeostatic proliferation contributes to HIV persistence by promoting the survival and proliferation of latently infected cells [13, 14]. Further highlighting the role of IL-7 in the expansion and maintenance of the viral reservoir, an ACTG sponsored trial (ACTG protocol number 5214; www.clinicaltrials.gov as # NCT00099671) demonstrated that IL-7, also leads to a 70% increase in the absolute numbers of CD4 T cells harboring integrated viral DNA [14], suggesting that this intervention would not be compatible with an HIV eradication strategy. IL-15, which also signals through STAT5, has also been demonstrated to induce homeostatic proliferation of CD4 T cell subsets [15–18]. Furthermore, recent *in vitro* and *ex vivo* studies with IL-15, the IL-15 superagonist (ALT-803) and IL-2 illustrated that not only did these γ-C cytokines increase viral reactivation, but they also primed the reservoir within CD4 T cells for recognition by autologous HIV-specific CD8 T cells [19].

Phosphorylation of STAT-5 (pSTAT5) is triggered following the engagement of IL-2, IL-7 or IL-15 cytokines with their receptors leading to pro-survival signaling and increased proliferation [15, 20, 21]. Given the presence of multiple binding sites for pSTAT5 within the HIV long terminal repeat (LTR) [22], IL-2, IL-7 and IL-15 enhanced viral expression from productively infected cells [14, 15, 20, 21, 23]. Interestingly, a dominant negative STAT5 inhibited Jak-induced HIV LTR activity and decreased productive HIV infection while overexpression of STAT5 enhanced virus production in unstimulated primary T cells [22]. Together, these events underscore the relationship between activation of the STAT5 pathway and production of HIV, including events that impact the establishment of latency, its maintenance, and /or expansion of the HIV viral reservoir [24–27].

Tofacitinib and ruxolitinib are two FDA-approved Jak inhibitors for long-term use for the treatment of rheumatoid arthritis, myelofibrosis, or polycythemia vera [28–30]. Ruxolitinib demonstrates potent inhibition of pro-inflammatory cytokines *in vivo*, including IL-6, IL-1α/β, and TNF-α [29–32], all of which have been shown to enhance HIV replication *in vitro* [33, 34]. Inhibition of Jak-STAT signaling by ruxolitinib was shown to significantly impede T cell homeostasis, reducing CD4 T cell numbers as well as decreasing numbers of T regulatory cells and activated (HLA-DR^+^) CD4 T cell populations after a few weeks of treatment [28]. Tofacitinib showed only small changes in CD3^+^, CD4^+^ and CD8^+^ counts and an increase in B cell counts after 24 weeks of treatment [29, 30, 35]. Attenuated activation and proliferation were not specific to CD4 T cells but were also reported for Natural Killer (NK) cells treated with ruxolitinib *in vitro* and also in ruxolitinib or tofacitinib treated patients where the number of mature NK cells was reduced [28, 35]. Ruxolitinib treatment was further shown to block monocyte–derived DC differentiation, DC-derived IL-12 production and activation marker expression triggered by exposure to lipopolysaccharide (LPS) [28], demonstrating the impact of Jak inhibitors on innate and adaptive immune responses.

We previously reported that ruxolitinib and tofacitinib blocked reactivation of HIV in a primary T cell latency model at physiologic concentrations, underscoring its potential to block HIV reservoir expansion and viral dissemination from latent stores [34]. Herein, we monitored the impact of these clinically approved and extensively evaluated Jak-STAT inhibitors on several stages of HIV persistence including seeding of bystander cells and HIV reactivation from latency. Overall, we show *ex vivo* and *in vitro* that these Jak inhibitors use several mechanisms to impede the seeding and maintenance of the HIV reservoir.

## Results

### Ex vivo markers of activation of the Jak-STAT pathway and of homeostatic proliferation are associated to the size of the HIV reservoir

We investigated the association between the Jak-STAT pathway and HIV persistence in a cohort (n = 37) of aviremic (<50 RNA copies/ml) ART-treated participants (S1 Table) to assess the *in vivo* relevance of Jak-STAT signaling in the establishment and maintenance of the HIV reservoir as monitored by frequencies of cells with integrated HIV DNA. Decreased CD4 numbers and immune activation are features of aberrant T cell homeostasis [13] and increased HIV reservoir size [36, 37], which was confirmed in the cohort studied here (S1 Table). We fit a linear model to identify univariate markers (Panel 1—activation markers, Panel 2—PD-1/IL-7R and Panel 3—STAT phosphorylation; S2 Table) associated with integrated HIV DNA (see methods). As previously described, the percent CD4+ T cells was significantly higher in immune responders (IR; median 34.7%) compared with non immune responders (NIR; median 22.4%) or recently treated (RT; median 24.7%) subjects (S3 Table) and increased integrated HIV DNA was associated with decreased frequencies of CD4+ T-cells (p = 0.019 [S2 Table]) [13, 14]. Indeed, we showed that increased levels of pSTAT5 expression in CD4 central memory (CM), transitional memory (TM) and in effector memory (EM) cells, all known to harbor HIV DNA, were associated with increased integrated HIV DNA (p = 0.043, 0.008, 0.001 respectively [S2 Table]). After adjusting for CD4 counts, CD4/CD8 ratio, nadir CD4 and pre-ART HIV-1 plasma RNA, CD4 CM, TM and EM pSTAT5 expression remained significantly associated with integrated HIV DNA (p = 0.031, 0.048 and 0.0048, respectively). As our cohort included immune responders (> 500 CD4 cells /mm^3^) and immune non responders (< 350 CD4 cells /mm^3^), successfully treated non-classified (SC NC) and recently treated subjects (< 1 year of treatment) (S1 Table), we determined the levels of pSTAT5 expression in each class. pSTAT5 in naïve CD4 cells was significantly lower in immune responders (median 1160 MFI) compared to immune non responders (median MFI 1500) or recently treated subjects (median 1523 MFI; p = 0.037 and 0.018 Wilcoxon rank test, respectively) (S17 Fig and S3 Table). There was no difference in STAT5 phosphorylation when comparing the different classes of subjects in CM, TM or EM CD4 cells, the subsets that encompass the bulk of HIV integrated DNA. The relationship between pSTAT5 and integrated viral DNA remains across immune responders and non-immune responders.

IL-7R engagement by its ligand leads to its internalization and to phosphorylation/activation of STAT5. Higher levels of surface IL-7R could reflect the lack of chronic receptor engagement, which would result in lower numbers of homeostatically proliferating cells and lower levels of the HIV reservoir [13, 14]. In line with this observation, we observed that increased surface expression of IL-7R on total CD4^+^ T-cells was associated with decreased integrated HIV DNA (p = 0.046, S2 Table), which was also significant in CD4 memory T cells (CD45RA-) (p = 0.032) and in CD4 CM cells (p = 0.032). For total CD4 T cells and CD4 memory T cells, IRs had significantly increased IL-7R levels (median 6916 MFI and 7163 MFI, respectively) compared with NIR (median 5314 MFI and 5288 MFI, respectively), RT (median 5536 MFI and 6091, respectively) or successfully treated non classified (median 6193 for CD4 memory T cells) classes of subjects; for the CD4 CM subset IL-7R levels in IRs (median 8647 MFI) was only significantly greater than ST NCs (median 7018 MFI, S3 Table). As cells from IRs are known to express lower levels of T cell activation and proliferation markers compared with NIRs [36, 37], increased expression of IL7R in IRs further supports our observation of decreased homeostatic proliferation and lower numbers of HIV infected cells in these subjects. We also monitored PD-1 expression, as it is up-regulated by γ-C receptor cytokines [38] and is a marker of cells undergoing homeostatic proliferation and the size of the HIV reservoir [37]. PD1 expression (MFI) on total CD4 (p = 0.027, S2 Table), naïve CD4 (p = 0.023), CD45RA- memory CD4 (p = 0.023), CD4 CM (p = 0.015) and CD4 TM (p = 0.037) cells was positively associated with increased integrated HIV DNA confirming previous reports [13, 14]. There was no difference in PD-1 levels (MFI) between IR and NIRs within this group of subjects however there was greater PD-1 levels on ST NC (median 9538 MFI) compared with IRs (median 7218 MFI, p = 0.048, S3 Table).

We also showed that an increase in frequencies of naïve CD4 T cells expressing Ki67 (p = 0.012, S2 Table), and cells expressing HLA-DR/CD38 (in total CD4 (p = 0.024), naïve CD4 (p = 0.008), CD45RA- memory CD4 (p = 0.03) and CD4 CM cells (p = 0.029)) were associated with increased frequencies of cells harboring integrated HIV DNA. The cells expressing HLA-DR/CD38 in naïve CD4 were correlated as well with increased frequencies of pSTAT5 positive CM, TM and EM subsets (p = 0.005, 0.027, 0.009 respectively). As observed in previous studies [36, 37], IRs had significantly lower levels of HLA-DR/CD38 in total CD4 T cells (median values for: IR—1.21%, NIR—2.36% and RT—2.22%), CD4 memory cells (CD45RA-) [median values for: IR– 1.83%, NIR—3.12%, RT– 3.55%] or CD4 CM cells (median values for: IR– 0.78%, RT– 1.38%) compared with NIRs and/or RT subjects; IRs also had lower levels of Ki67 in naive CD4 T cells (median 0.7%) than NIR (median 0.18%) (S3 Table). Heightened frequencies of cells with integrated HIV DNA were also associated with a decrease in frequencies of CD25+ cells in total CD4 (p = 0.017, S2 Table), memory (CD45RA-) CD4 (p = 0.002), as well as in CM (p = 0.0002) and TM (p = 0.009). In contrast to HLA-DR/CD38 and Ki67 expression, IRs had significantly greater levels of CD25 on CD4 T cells (median values for: IR– 40%, RT– 32%, ST NC– 29%), CD4 memory cells (CD45RA-) [median values for: IR– 56%, NIR– 42%, RT– 44%, ST NC– 42%], and CD4 CM cells (median values for: IR– 57%, NIR– 50%, ST NC– 46%) compared with NIR, RT and/or ST NC (S3 Table). These results demonstrated that up-regulated levels of pSTAT5 were associated with deregulated homeostatic proliferation and the size of the HIV reservoir and immune activation.

Within each of the panels (panel 1 –activation markers, panel 2 –PD-1/IL-7R or panel 3 –STAT phosphorylation), we next identified the combination of the univariate markers that could best predict the size of the HIV reservoir by building a multivariate regression model using feature selection [39] (see methods; Table 1). The activation markers panel showed that frequencies of total CD4+ T-cells, of naïve CD4+ cells expressing HLA-DR/CD38 (markers of immune activation) and Ki67, and of CD4+ CM cells expressing CD25 best predicted the size of the HIV reservoir (F-statistic p-value = 0.004 –Table 1). Within the PD-1/IL-7R panel, expression (MFI) of PD-1 by CM and TM cells, and of IL7R by CM cells best predicted the size of the HIV reservoir (F-statistic p-value = 0.04), while the STAT phosphorylation panel, showed only STAT5 phosphorylation on CD4+ EM cells as a predictor of the size of the HIV reservoir (F-statistic p-value = 0.001).

**Table 1:** Multivariate model of the *ex vivo* Jak-STAT pathway markers that best predict the size of the HIV reservoir.

Panel	Cell Type^a^	Coefficient of Regression^b^	F-statistic
**1 –Activation Markers**	%CD4+ cells	-0.172	0.004
	%CD4+ HLADR/CD38+ Naïve cells	0.03	
	%CD4+ Ki67+ Naïve cells	0.107	
	%CD4+ CD25+ CM cells	-0.444	
**2—PD-1/IL-7R**	MFI CD4+ PD1+ CM cells	0.184	0.041
	MFI CD4+ IL7R+ CM cells	-0.273	
	MFI CD4+ PD1+ TM cells	0.151	
**3—STAT phosphorylation**	MFI CD4+ pSTAT5+ EM cells	0.528	0.001

^a^ CM–central memory, TM–transitional memory, EM–effector memory

^b^ Multivariate regression model using feature selection (see methods)

These multivariate models further confirm results of the univariate analyses showing the significant association between immune activation, T cell homeostasis and the HIV reservoir. These results provide further rationale for the use of interventions that target the Jak-STAT pathway *i*.*e* inhibitors of the Jak-STAT pathway that are known to inhibit immune activation and γ-C cytokine induced proliferation which could impact the size of the HIV reservoir.

### Ruxolitinib and tofacitinib inhibit cytokine-induced STAT5 phosphorylation and Bcl-2 expression

**Fig 1:**
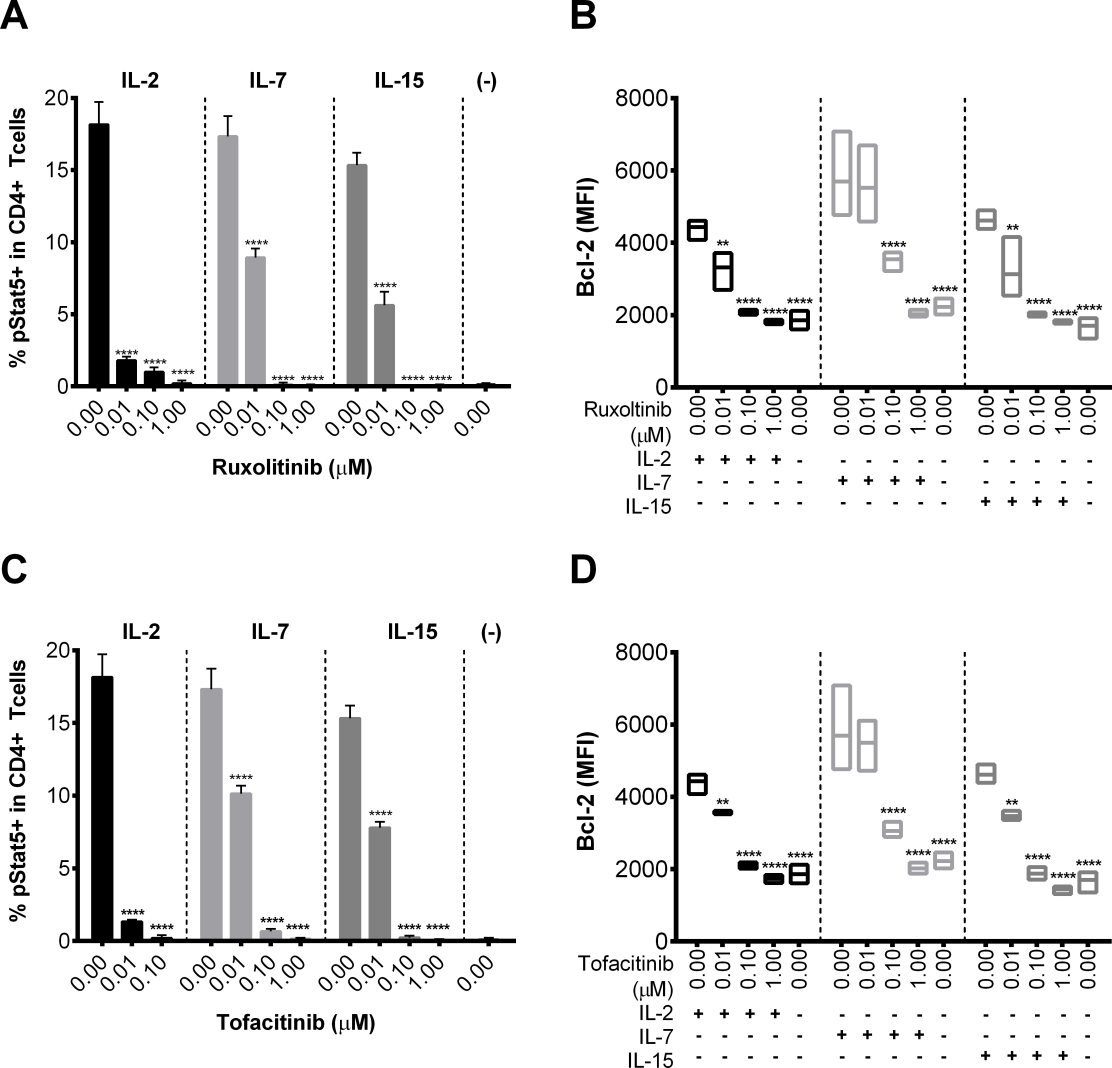
Jak inhibitors block cytokine-induced STAT5 phosphorylation and Bcl-2 expression. STAT5 phosphorylation (% in CD4+ T cells) or Bcl-2 expression (MFI in CD4+ T cells) was measured by flow cytometry in PBMC isolated from HIV negative donors and stimulated for 15 min (pSTAT studies; A, C) or 6 days (Bcl-2 studies; B, D) with IL-2 (left panels), IL-7 (middle panels) and IL-15 (right panels) (n = 3) and increasing concentrations (0.01, 0.1, and 1.0 μM) of ruxolitinib or tofacitinib (A-D). 0.0 μM represents the average of all assays completed using % DMSO equivalent to Jak inhibitor concentrations. (-) indicates no cytokine was added. Error bars represent S.E.M. and statistical significance determined by two-way ANOVA followed by Sidak’s multiple comparison post-test: **p < 0.01 and ****p < 0.0001.

We monitored, *in vitro*, STAT5 phosphorylation following γ-C receptor cytokine stimulation in total CD4 T cells and in the different memory subsets known to harbor the HIV reservoir in the presence or absence of the Jak inhibitors ruxolitinib and tofacitinib (Fig 1, S1 Fig and S4 Table). As expected, ruxolitinib significantly (p < 0.001) reduced IL-2, IL-7, and IL-15 induced pSTAT5 expression (Fig 1A) in a dose dependent fashion. Activity of ruxolitinib on STAT5 phosphorylation triggered by all three cytokines was completely abrogated at concentrations ≥ 0.1 μM (Fig 1A) although this inhibitor had a significantly stronger impact on IL-2 induced CD4^+^ STAT5 phosphorylation (10- fold reduction) when compared to IL-7 and IL-15 at 0.01 μM (1.9- and 2.7- fold reduction, respectively) (Fig 1A). These results were confirmed with tofacitinib where we also observed a similar dose dependent inhibition of the frequencies of pSTAT5^+^ cells (Fig 1C; p < 0.001). Importantly, ruxolitinib and tofacitinib completely abrogated frequencies of pSTAT5^+^ cells exposed to IL-2, IL-7 and IL-15, compared with DMSO for concentrations > 0.1 μM (p < 0.01) in all CD4 memory subsets (T_CM_,T_TM_ and T_EM_) all known to harbor HIV integrated DNA (S1 Fig and S4 Table) [13]. Both Jak-STAT inhibitors showed a stronger impact on IL-2 induced STAT5 phosphorylation in all memory subsets as we had observed in total CD4^+^ T cells (S1 Fig). A linear regression model to study the association between the frequencies of pSTAT5^+^ cells within each CD4^+^ memory subset with a range of Jak-STAT inhibitor concentrations showed a negative correlation between these two variables, *i*.*e*. percent pSTAT5^+^ cells significantly decreased with increasing concentrations of ruxolitinib, specifically in CM and TM cells when exposed to IL-2 (p-values < 0.05) and with increasing concentrations of tofacitinib, in CM and TM cells when exposed to IL-15 and IL-2, while in the EM cells when exposed to IL-7 (at p-values < 0.05) (S4 Table and S1 Fig). These inhibitors also significantly (p < 0.01) decreased IFN-α stimulated STAT5 phosphorylation in CD4^+^ T-cells, IFN-α induced STAT1 phosphorylation in CD14^+^ monocytes and CD4^+^ T-cells and IL-10 induced STAT3 phosphorylation in CD14^+^ monocytes and CD4^+^ T-cells (S2 Fig) in a dose dependent fashion highlighting a possible role for these inhibitors in lowering the levels of hyper immune activation.

We also monitored for the upregulation of Bcl-2 expression triggered by these homeostatic cytokines; Bcl-2 is a transcriptional target of STAT5, which enhances the survival of cells exposed to IL-2, IL-7 and IL-15 [20, 40]. Both ruxolitinib (Fig 1B) and tofacitinib (Fig 1D) significantly (p < 0.01) reduced IL-2, IL-7, or IL-15-induced Bcl-2 levels (MFI) of expression in a dose dependent fashion to levels similar to the unstimulated control. Significant reduction of Bcl-2 expression (p < 0.01) was achieved at concentrations of the Jak-STAT inhibitors as low as 0.01 μM for IL-2 and IL-15 induced Bcl-2 expression (1.3- and 1.5- fold reduction, respectively). IL-7 induced Bcl-2 regulation required higher concentrations of these inhibitors as inhibition of IL-7 induced Bcl-2 levels (MFI) was only observed at concentrations ≥ 0.1 μM of ruxolitinib and tofacitinib (Fig 1B and 1D). Using a linear regression model that included Bcl-2 levels (MFI) within each CD4^+^ memory subset as the dependent variable and Jak-STAT inhibitor concentration as the independent variable, we showed that Bcl-2 expression (MFI) also significantly (p < 0.05) decreased with increasing concentrations of ruxolitinib or tofacitinib in all memory subsets (CM, TM and EM) upon exposure to IL-2, IL-15 and IL-7 (S4 Table and S1 Fig).

Altogether these results confirmed the inhibitory impact of ruxolitinib or tofacitinib on the activation of the Jak-STAT pathway in all CD4+ memory T cell subsets known to harbor the HIV reservoir.

### Ex vivo and in vitro inhibition of HIV replication by ruxolitinib and tofacitinib

**Fig 2:**
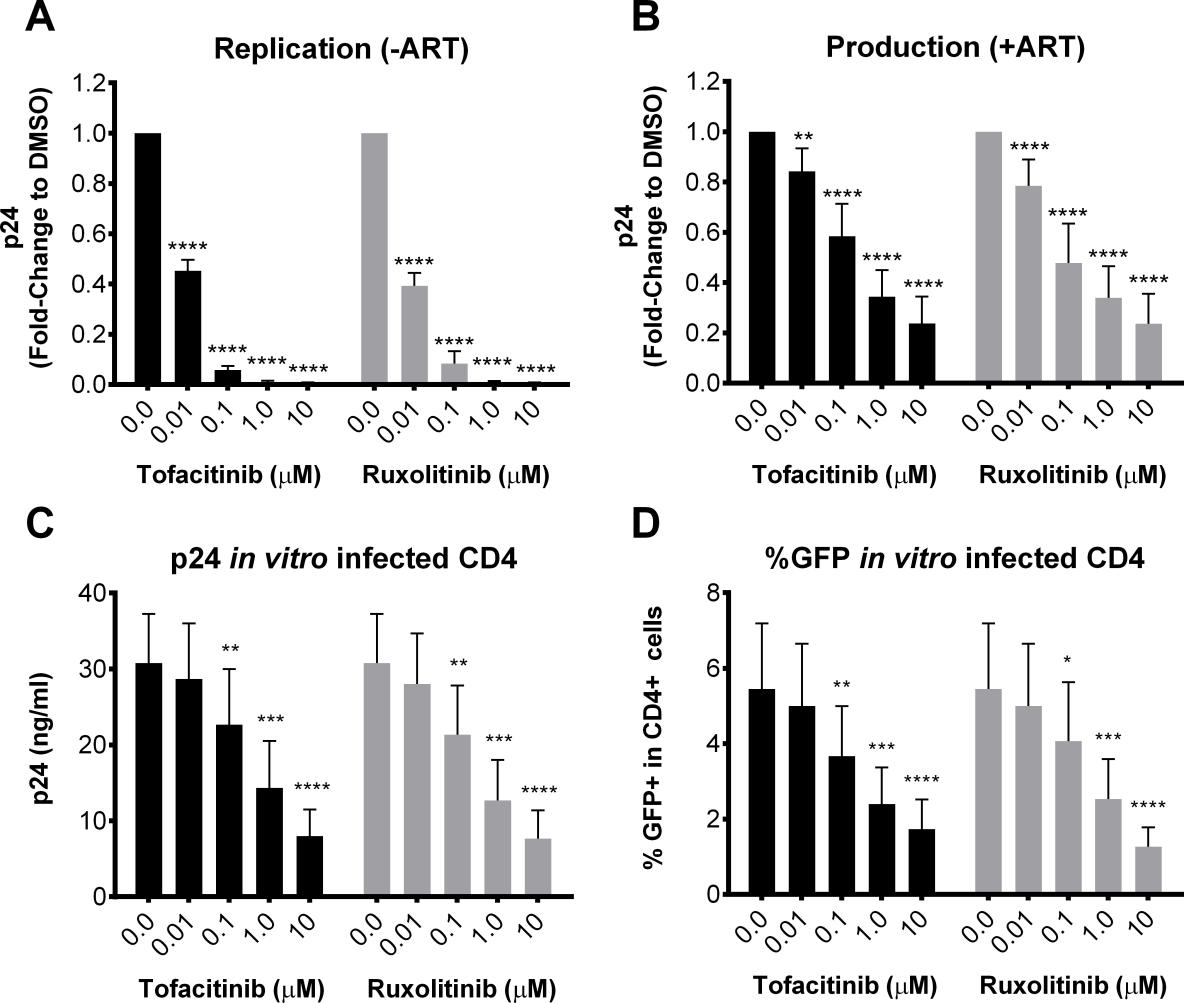
Jak inhibitors block HIV-1 replication and production *ex vivo* and *in vitro*. Viral production was measured by ELISA p24 in cell-free supernatants of enriched CD4^+^ T cells isolated from 5 viremic donors and stimulated for 6 days with anti-CD3/28 in the presence of increasing concentrations of Jak inhibitors without (A) or with (B) ART. Viral production was measured by ELISA p24 in cell-free supernatants of *in vitro* infected CD4^+^ cells after 6 days culture in the presence of increasing concentrations of Jak inhibitors (C) and by the frequency of HIV-GFP+ expressing CD4+ cells after 3 day culture (D). To account for inter-patient variability in baseline values, results are reported as the fold change *versus* DMSO controls. 0.0 μM represents the average of all assays completed using % DMSO equivalent to Jak inhibitor concentrations. Error bars represent S.E.M. and statistical significance determined by two-way ANOVA followed by Sidak’s multiple comparison post-test: *p < 0.05, **p < 0.01, ***p < 0.001 and ****p < 0.0001.

Our group previously demonstrated that ruxolitinib and tofacitinib confer submicromolar anti-HIV-1/2 activity in human PBMCs and in macrophages without demonstrable toxicity [34], however further mechanistic information about how these drugs modulate key events involved in the expansion and maintenance of the HIV viral reservoir have not been explored. Herein we show that both inhibitors significantly (p < 0.0001) reduced p24 production by CD4^+^ T cells isolated from viremic donors and stimulated with CD3/CD28 when compared to DMSO controls (Fig 2A and 2B and S3 and S4 Figs for data on individual donors). This inhibition was observed when tested in the absence of ART (to observe the effect of the Jak inhibitors on viral replication and *de novo* infection of cells) and in the presence of ART (to observe the effect of the Jak inhibitors when spreading of HIV infection is inhibited, hence on viral production). These results showed that Jak inhibitors prevented uninfected primary CD4 T cells from HIV infection (described in detail under bystander infection assay) and inhibited the production of HIV from infected cells (ruxolitinib antiviral potency with ART; EC_50_ 0.17 μM; EC_90_ 6.2 μM; ruxolitinib antiviral potency without ART; 0.007 μM; EC_90_ 0.26 μM).

Since ruxolitinib and tofacitinib decreased γ-C-cytokine induced Bcl-2 expression (Fig 1), we monitored cell viability of CD4^+^ T cells isolated from viremic donors and cultured for 6 days with CD3/CD28 and increasing concentrations of Jak inhibitors. Decreased viability was observed in 3 of the 5 donors at 0.1 μM or greater ruxolitinib or tofacitinib (S15 Fig, Panel A); using a linear regression model, addition of either inhibitor to anti-CD3/CD28 stimulated cell cultures led to significantly lower frequency of viable cells (%AnnexinV-LIVE/DEAD-) compared to DMSO taking into account inhibitor concentration and adjusting for donor. In contrast, we did not observe a loss in viability of *in vitro* infected cells after 3 day culture with Jak inhibitors plus ART (S15 Fig, Panel B); and no loss in viability was observed after 6 day culture of uninfected cells treated with IL-2 or IL-7 (n = 3) and in 2 of 3 uninfected donors treated with IL-15 in the presence of Jak inhibitors (S15 Fig, Panel C). As HIV-1 infected subjects are known to express lower levels of Bcl-2 [41], decreased viability as a result of inhibition of Bcl-2 expression may be a potential mechanism of Jak inhibitors on blocking viral persistence along with HIV infection in CD4 cells from viremic donors.

The inhibition of viral replication and production mediated by ruxolitinib and tofacitinib *ex vivo* was confirmed in an *in vitro* HIV infection model using a CXCR4-tropic, GFP tagged virus (eGFP NL4-3 replication competent HIV-1 reporter virus) that was used to identify productively infected cells by flow cytometry. At day 6 post infection, ruxolitinib and tofacitinib (0.1, 1.0, and 10 μM) significantly (p < 0.05) reduced extracellular p24 production (Fig 2C) as well as the frequency of HIV-GFP^+^ CD4^+^ cells (Fig 2D). Both inhibitors significantly (p < 0.01) decreased CCR5 surface expression on CD4 T cells that was upregulated as a result of anti-CD3/CD28 stimulation in viremic subjects (n = 5) whereas the levels of CXCR4 were not impacted by the addition of these inhibitors (S5 and S6 Figs). Since CXCR4 expression remained unchanged, decreased viral production and viral spread may be mediated through a mechanism beyond entry such as reduced T-cell activation (S16 Fig). As CCR5 dependent viral strains are mostly prevalent *in vivo* [42–44], these results indicate that inhibition of anti-CD3/28 induced CCR5 expression by ruxolitinib could have an impact on viral spread and dissemination in an infected untreated host [42, 43] and specifically in tissues from ART treated subjects where HIV replication still prevails in spite of the presence of ART [33, 45].

Since γ-C cytokines promote the activation of the Jak-STAT pathway (Fig 1) [8, 9, 11], paracrine or autocrine inhibition of IL-2, IL-7, and IL-15 induced signaling by ruxolitinib could be in part responsible for the inhibitory effects exerted by this compound on p24 production. Addition of an exogenous source of IL-7 (30 ng/ml) in *ex vivo* CD4 T cell cultures from viremic donors reversed the block by ruxolitinib at an antiviral EC50 concentration of 33 nM conferred on extracellular HIV production (p < 0.01), measured by p24 after 6 days in culture (S7 Fig; *n* = 4). More specifically, exogenous IL-7, which signals largely through STAT5, reverses latency, underscoring the link between IL-7, the ability to control latency, and STAT5-mediated signaling.

### Jak inhibitors reduce the frequency of cells harboring integrated HIV viral DNA ex vivo and block IL-15 induced reactivation of latent HIV ex vivo

**Fig 3:**
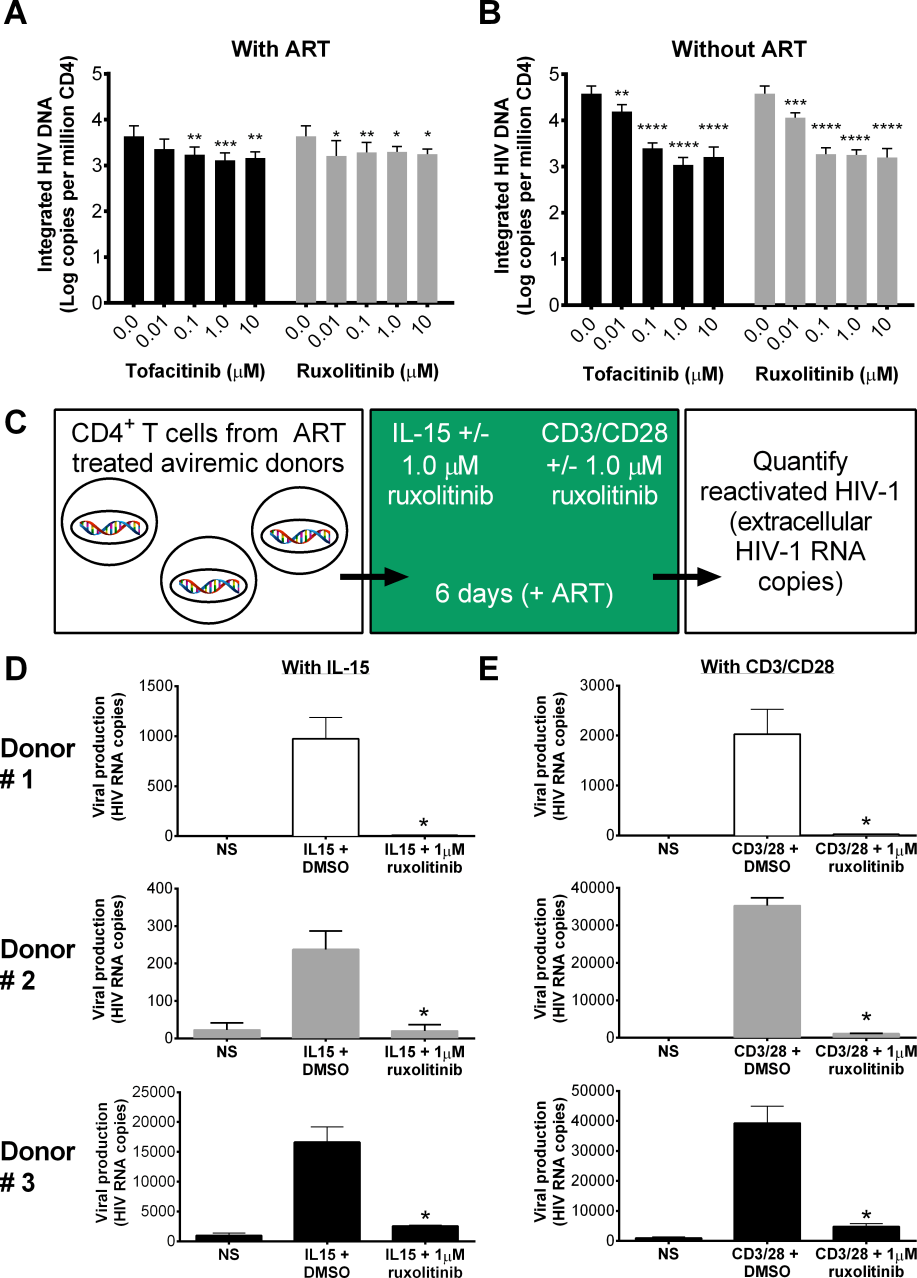
Jak inhibitors reduce frequency of cells harboring integrated viral DNA and IL-15-induced reactivation of latent HIV-1 in CD4 T cells. CD4 T cells were isolated from viremic donors and incubated with CD3/CD28 plus 0.01, 0.1, 1.0 or 10 μM of Jak inhibitors with or without EC_99_ of ART (180 nM zidovudine, 100 nM efavirenz, 200 nM Raltegravir) (A and B). After six days, integrated viral DNA was quantified using ultra sensitive Alu PCR *versus* DMSO controls (n = 5). 0.0 μM represents the average of all assays completed using % DMSO equivalent to Jak inhibitor concentrations. Error bars represent S.E.M. and statistical significance determined by two-way ANOVA followed by Sidak’s multiple comparison post-test: *p < 0.05, **p < 0.01, ***p < 0.001 and ****p < 0.0001 for A and B. In panel C-E, memory CD4^+^ T cells were isolated from ART treated aviremic donors (n = 3), activated with 10 ng/ml IL-15 (panel D) or CD3/CD28 (panel E) and maintained with or without 1 μM ruxolitinib in the presence of ART. Six days post reactivation, extracellular viral RNA copies were quantified by qRT-PCR (*p < 0.01, one-way ANOVA).

We monitored the frequency of cells harboring integrated viral DNA in cultures of T cells obtained from viremic donors that were activated by T cell receptor (TCR) engagement. These experiments aimed at measuring the impact of ruxolitinib on the maintenance of the existing reservoir as cultures were generated in the presence of ART. Cultures were also performed in the absence of ART to measure the seeding of the viral reservoir, as under these conditions infected cells that can produce virions will infect new T cells. Ruxolitinib and tofacitinib significantly (p < 0.05) decreased the frequency of cells with integrated DNA in cultures of T cells activated by TCR in the presence or absence of ART (Fig 3A and 3B) with doses of ruxolitinib as low as 0.01 μM.

IL-15, which signals through STAT5, also regulates memory T cell homeostasis [17, 18, 21], and could be involved in modulating the HIV reservoir size by promoting the persistence of cells with integrated DNA or by enhancing HIV reactivation and dissemination. Since ruxolitinib and tofacitinib inhibited STAT5-mediated signaling triggered by IL-7 and IL-15 (Fig 1) we sought to define the impact of ruxolitinib on IL-15 induced reactivation of latent HIV in CD4 T cells from aviremic subjects (Fig 3C–3E). Memory CD4^+^ T cells were isolated from ART treated aviremic donors (n = 3), activated with a concentration of IL-15 that is known to activate Jak-STAT signaling (10 ng/ml) (Fig 3D) or CD3/CD28 (Fig 3E) and maintained with or without 1 μM ruxolitinib in the presence of ART. We showed in preliminary experiments that 10 ng/ml of IL-15 triggered optimal STAT5 phosphorylation and viral reactivation. Extracellular viral RNA copies were quantified by qRT-PCR six days post IL-15 activation of purified memory CD4 T cells. Indeed, IL-15 increased reactivation of latent HIV (200 to > 1,000-fold *versus* non stimulated controls), although to a lesser extent when compared to the CD3/28 control (> 30,000 fold *versus* non stimulated controls; Fig 3C–3E). However, ruxolitinib was found to significantly reduce (p < 0.01) IL-15 and CD3/28 induced reactivation of HIV from latently infected cells, resulting in values similar to unstimulated controls. Additionally, our results demonstrated that ruxolitinib and tofacitinib decreased anti-CD3/28 induced T cell proliferation (dilution of Cell Trace Violet; S8 and S9 Figs) and activation (CD25, CD38/HLA-DR and PD-1 expression; S8 Fig and S10–S12 Figs) and more importantly, led to a decrease in the frequency of p24^+^ cells (Fig 2) as well as a reduction in cells harboring integrated HIV provirus (Fig 3) and decreased IL-15 induced HIV reactivation (Fig 3). Each of these events mechanistically signals through STAT5, further highlighting the role of STAT5, and subsequent block by Jak inhibitors, in controlling these key events that drive viral persistence. Altogether these results indicate that Jak-STAT inhibitors can negatively impact *de novo* seeding and the maintenance of the HIV reservoir.

### Ruxolitinib inhibits HIV bystander infection in human PBMCs

**Fig 4:**
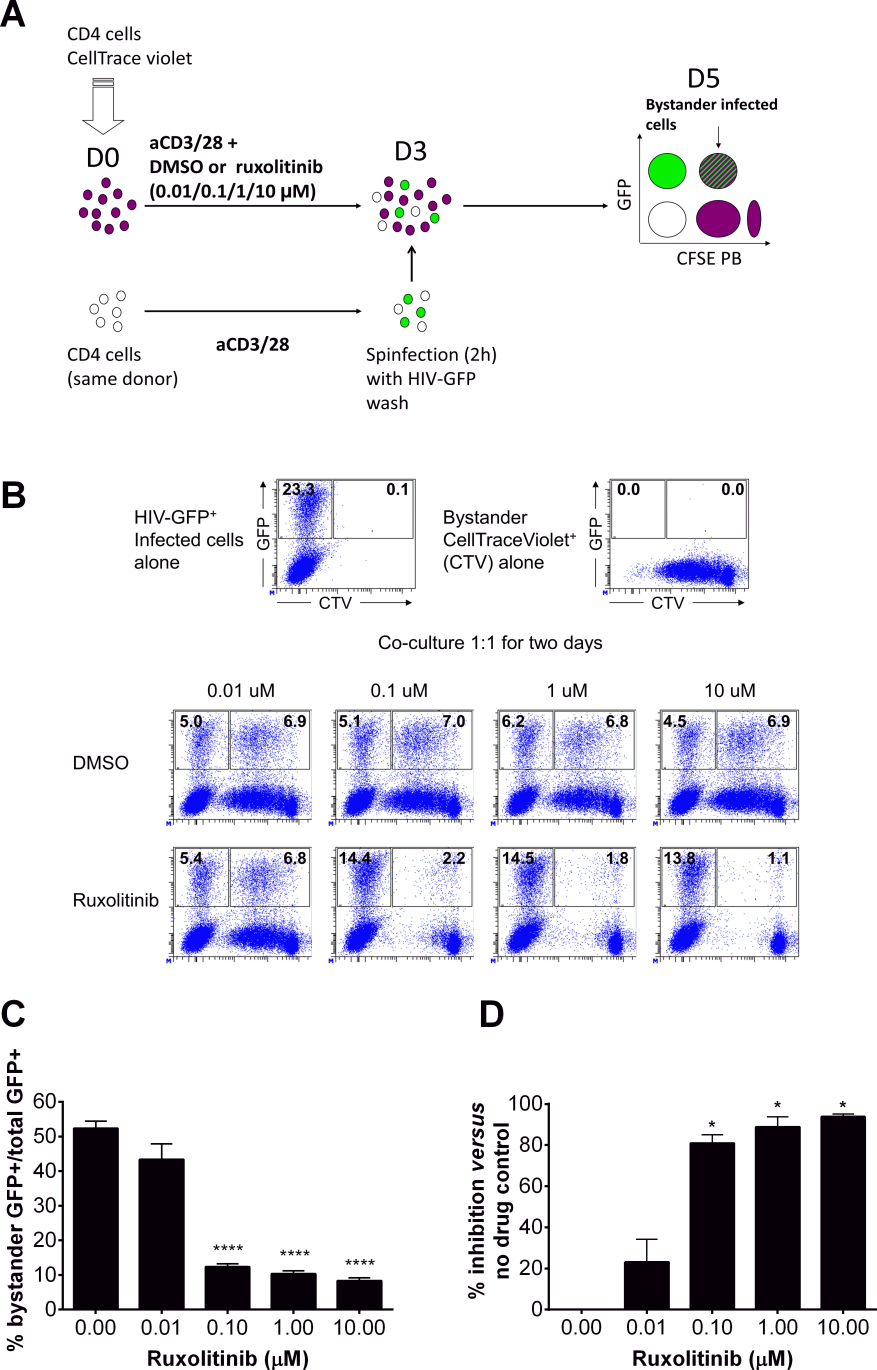
Ruxolitinib inhibits bystander infection. Uninfected CD4^+^ T cells were incubated with or without cell trace violet (CTV) dye. Cells with CTV dye were stimulated with CD3/CD28 and various concentrations of ruxolitinib or DMSO for 3 days (A, top). Cells without CTV dye were incubated with CD3/CD28 for 3 days followed by a 2 hours spinoculation with a replication competent eGFP Nl4-3 X4 HIV-1 (A, bottom). After spinocualtion on Day 3, both cultures (traced and untraced) were co-incubated for two days in the absence of ruxolitinib. Representative dot plots for bystander infection quantification are demonstrated in panel B. Ruxolitinib inhibits bystander infection (GFP and CTV double positive) of uninfected bystander cells (CTV^+^) in a dose dependent manner (B and C, n = 3). Ruxolitinib blocks proliferation (CTV-lo) of bystander cells in a dose dependent manner with all concentrations tested (D). 0.0 μM represents the average of all assays completed using % DMSO equivalent to Jak inhibitor concentrations. Error bars represent mean with S.E.M (C) or mean with standard deviation (D) and statistical significance determined by two-way ANOVA followed by Sidak’s multiple comparison post-test (C; ****p < 0.0001) or a two-tailed paired T test (D; *p < 0.005).

The impact of ruxolitinib on the magnitude of the HIV reservoir could result from the inhibition of TCR triggered CD4 T cell activation and proliferation (S8–S12 Figs). It could also result from the direct antiviral activity of the Jak-STAT inhibitors (Fig 2A and 2B). Finally, decreased frequencies of cells harboring HIV integrated DNA could result from the diminished infection of bystander activated T cells due to the effects of these compounds on T cell activation and viral replication. We sought to determine if the presence of ruxolitinib could prevent the transfer of infectious viral particles to uninfected bystander cells or upon the formation of a virological synapse [44]. We developed an *in vitro* model to assess the impact of ruxolitinib on infection of bystander cells (schematic: Fig 4A; representative dot plots: Fig 4B) where bystander cells were labeled with cell trace violet (CTV^+^) and stimulated with CD3/28 in the presence of increasing concentrations of ruxolitinib to inhibit their activation. Unlabeled cells, (CTV^-^), were stimulated with CD3/28 in the absence of ruxolitinib, prior to infection with eGFP NL4-3. Infected CTV-negative cells and bystander CTV-positive cells were then co-cultured for two days to determine the number of ruxolitinib treated bystander cells susceptible to infection by eGFP virus (CTV^+^GFP^+^). As expected Ruxolitinib blocked proliferation (CTV-lo) of bystander cells (range 23% - 94%; p < 0.01 compared to no drug controls; paired t test) at all concentrations tested (Fig 4D). Cultures that did not include ruxolitinib led to the infection of fifty percent of bystander CTV^+^ cells (CTV^+^GFP^+^ cells) while addition of ruxolitinib led to a 75–80 percent reduction (p < 0.001) in CTV^+^GFP^+^ infected bystander cells. Our results show that ruxolitinib inhibition of Jak-STAT signaling impacts the susceptibility of uninfected cells to infection by HIV-1 at concentrations ≥ 0.01 μM (Fig 4B: representative dot plots, and Fig 4C: graphical representation) which are comparable to the *ex vivo* EC_50_ of 0.007 μM for ruxolitinib in CD4 T cells from viremic donors and well below the EC_90_ of 0.26 μM (Fig 2A). These findings suggest that Jak-STAT inhibitors could synergize with ART and decrease seeding of the HIV reservoir [34] in uninfected bystander cells.

### Ruxolitinib does not inhibit TCR downstream proximal signaling and function

**Fig 5:**
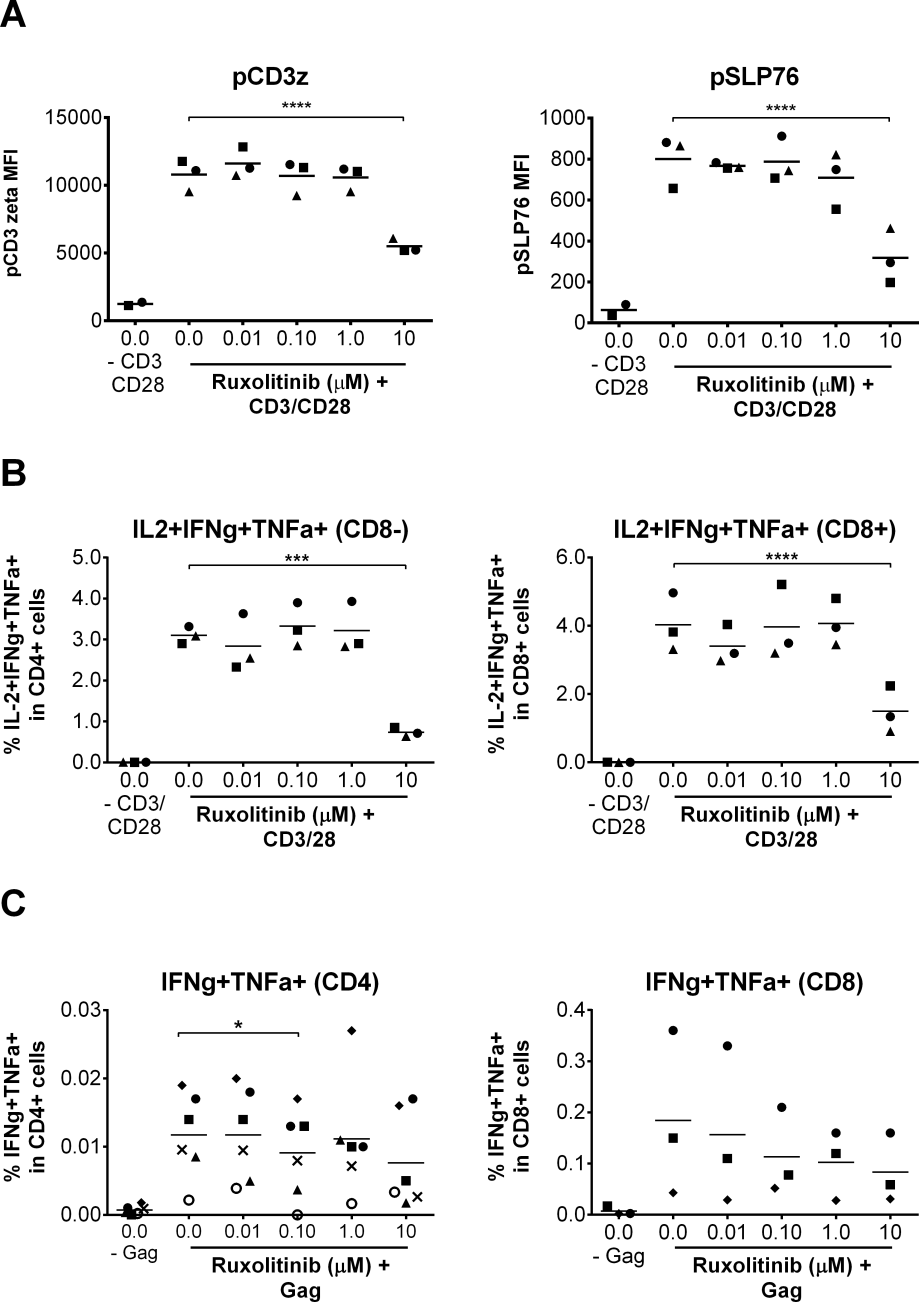
Ruxolitinib does not inhibit normal TCR function and signaling that is independent of HIV-1 infection. **(A)** Mean CD3 zeta and SLP76 phosphorylation (MFI in CD4 cells) as quantified by flow cytometry in CD4^+^ cells isolated from HIV negative donors and stimulated with anti-CD3/CD28 in the presence of increasing concentrations of Ruxoltinib *versus* DMSO treated control cells (n = 3). Statistical significance was determined by an upaired t-test corrected for multiple comparisons using the Holm-Sidak method. **(B)** Mean cytokine production (% of IL-2^+^, TNF-α^+^ and IFN-γ^+^ triple positive cells) in CD3+CD8- cells or CD3+CD8+ cells as measured by flow cytometry in PBMC isolated from HIV negative donors and stimulated for 6 hr with aCD3/CD28, Brefeldin A (5 μg/ml) and increasing concentrations of Ruxoltinib *versus* DMSO treated cells (n = 3). Statistical significance for **(B)** determined by two-way ANOVA followed by Sidak’s multiple comparison post-test: *p < 0.05, **p < 0.01, ***p < 0.001 and ****p < 0.0001. **(C)** Mean cytokine production (% of TNF-α^+^IFN-γ^+^ double positive cells) in CD3+CD4+ cells (n = 6) or CD3+CD8+ cells (n = 3) as measured by flow cytometry in PBMC isolated from stably treated, HIV positive donors and stimulated for 6 hr with 1 μg/ml gag-peptide, Brefeldin A (5 μg/ml) and increasing concentrations of Ruxoltinib *versus* DMSO treated cells. 0.0 μM represents the average of all assays completed using % DMSO equivalent to Jak inhibitor concentrations. Statistical significance for **(C)** determined by paired Wilcoxon rank sum test.

An HIV cure will involve the elimination of residual persistently infected cells by immune effector mechanisms that include HIV specific T cells. Therefore therapeutic strategies such as Jak-STAT inhibition should not have an impact on T cell effector functions. We monitored the impact of ruxolitinib on the early and late signaling events downstream of TCR activation (n = 3). Fig 5A evaluated the effect of ruxolitinib on phosphorylation of SLP76 and CD3 zeta, two early events of the T cell receptor-signaling cascade [16, 46]. Fig 5A shows that ruxolitinib did not significantly alter CD3 zeta and SLP76 phosphorylation at all physiological concentrations (steady-state plasma concentrations found *in vivo* for all doses of ruxolitinib) tested (0.01, 0.1, and 1.0 μM) (S13 Fig- gating strategy and Fig 5A).

We next monitored the impact of ruxolitinib on late TCR signaling events such as the capacity of CD4^+^ T cell to produce effector cytokines (TNF-α, IL-2 and IFN-γ) when stimulated with CD3/CD28 (Fig 5B and S14 Fig) Ruxolitinib concentrations of 0.01, 0.1 and 1.0 μM did not alter cytokine production of IL-2, TNF-α, or IFN-γ single positive cells or IL2+TNFα+IFNγ+ polyfunctional CD4+ and CD8+ T-cells, as quantified by intracellular flow cytometry (Fig 5B and S14 Fig). Decreased cytokine production was observed only at 10 μM, which is above the C_max_ and steady-state concentrations of ruxolitinib found *in vivo* [31]. Similarly, upstream events of TCR signaling and production of TNF-α, IL-2 and IFN-γ were inhibited only upon addition of a supra-physiological concentration (10 μM) of ruxolitinib, [32]. When we monitored cytokine production after gag peptide stimulation of PBMCs from stably treated, HIV-infected subjects, statistically significant differences in gag-specific antigenic responses were observed only at 10 μM ruxolitinib for TNF-α and at 0.1 μM for TNFα+IFNγ+ in CD4+ cells (Fig 5C and S18 Fig). There were no significant differences in gag-specific responses in CD8+ T-cells at the different concentration of ruxolitinib, however only 3 of the 6 subjects tested responded to gag-peptide stimulation and two subjects demonstrated decreased cytokine production at ruxolitinib concentrations ≥ 0.1 μM (Fig 5C and S18 Fig). These results demonstrate that ruxolitinib does not inhibit initial TCR function in both CD4 and CD8 T-cells nor HIV specific responses in CD4 T-cells with some donor-specific responses in CD8 T-cells, highlighting the ability of ruxolitinib to specifically inhibit signal transduction pathways that alter T cell proliferation and expansion of the HIV reservoir without interfering with the development of effector antiviral T cell functions.

### Pharmacokinetic simulation for 10 mg and 20 mg bis in die (twice daily; bid) dosing of ruxolitinib in humans

**Fig 6:**
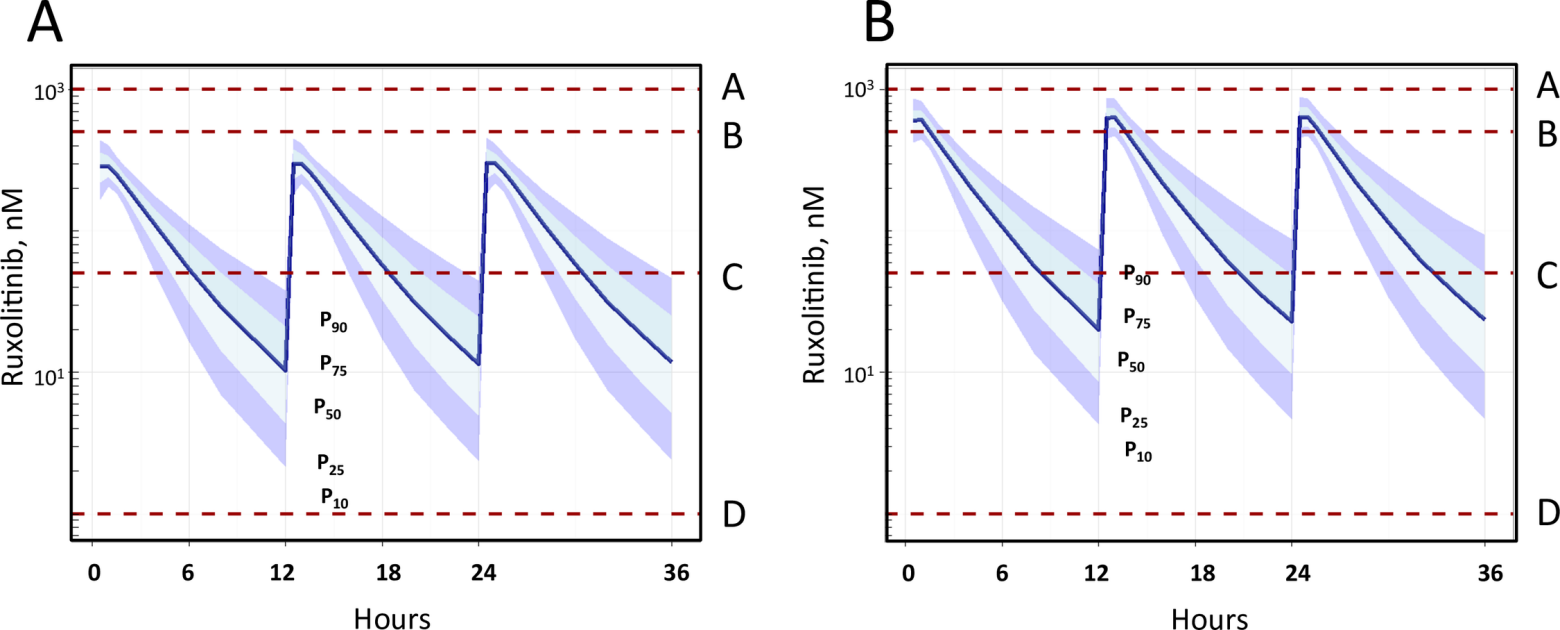
Pharmacokinetic simulation for 10 mg and 20 mg bid dosing of ruxolitinib demonstrates that anti-HIV effects occur at physiologically relevant concentrations observed in humans. Simulation of *in vivo* pharmacokinetics of 10 mg (A) or 20 mg (B) bid ruxolitinib demonstrated that all pro-HIV events that were inhibited by ruxolitinib in *vitro* occur at or below concentrations within the steady state plasma concentrations observed *in vivo* for 10 mg bid (A), and 20 mg bid (B). Dotted lines denote IC_50_ at which ruxolitinib confers inhibition *in vitro*, and notations of A-D denote: CD3 zeta and pSLP76, A; inhibition of Bcl-2 activation, B; inhibition of maintenance and expansion of the T cell reservoir, and antiviral potency against chronic and acute infection, C; inhibition of proliferation/activation (CD38/HLADR, PD1), down regulation of CCR5, inhibition of pSTAT5 by IL-2, IL-7, IL-15, inhibition of bystander infection, D.

Pharmacokinetic simulations were performed using reported plasma drug levels [31, 32] to determine if the plasma concentrations of drug during either 10 mg (Fig 6A), or 20 mg (Fig 6B) bid oral ruxolitinib treatment, could be correlated with *in vitro* efficacy. To assess PK/PD (pharmacokinetics/pharmacodynamics) relationship relative to the *in vitro* and *in vivo* efficacy reported herein, baseline population pharmacokinetics (PPK) model parameters without explicit patient covariates were used to construct a PPK model. Our model used δ^2^ as the ↔ subject variance (IIV) for that PK parameter, and ⌠^2^ = residual variance (within subjects). Log-normal error structure was used and 1,000 theoretical participants administered 10 mg ruxolitinib twice per day were modeled. Resulting computed percentile (P_10_, P_25_, P_50_ P_75_, P_90_) plasma concentrations *versus* time were used to mimic *in vivo* doses of 10 and 20 mg bid, which represent the low and high FDA-approved doses.

The model presented in Fig 6 shows that the pharmacodynamic effects (anti-HIV effects on viral reservoirs, antiviral, and HIV-induced activation/proliferation) of ruxolitinib falls within the C_max_ and steady-state range for all FDA approved doses of the drug (dotted lines; 0.01–1.0 μM: Fig 6), highlighting that the concentrations required to inhibit these pro-HIV events are equivalent *in vivo* for individuals taking ruxolitinib, even for the 10 mg bid dosing, which represents the lowest effective dose in humans.

## Discussion

**Fig 7:**
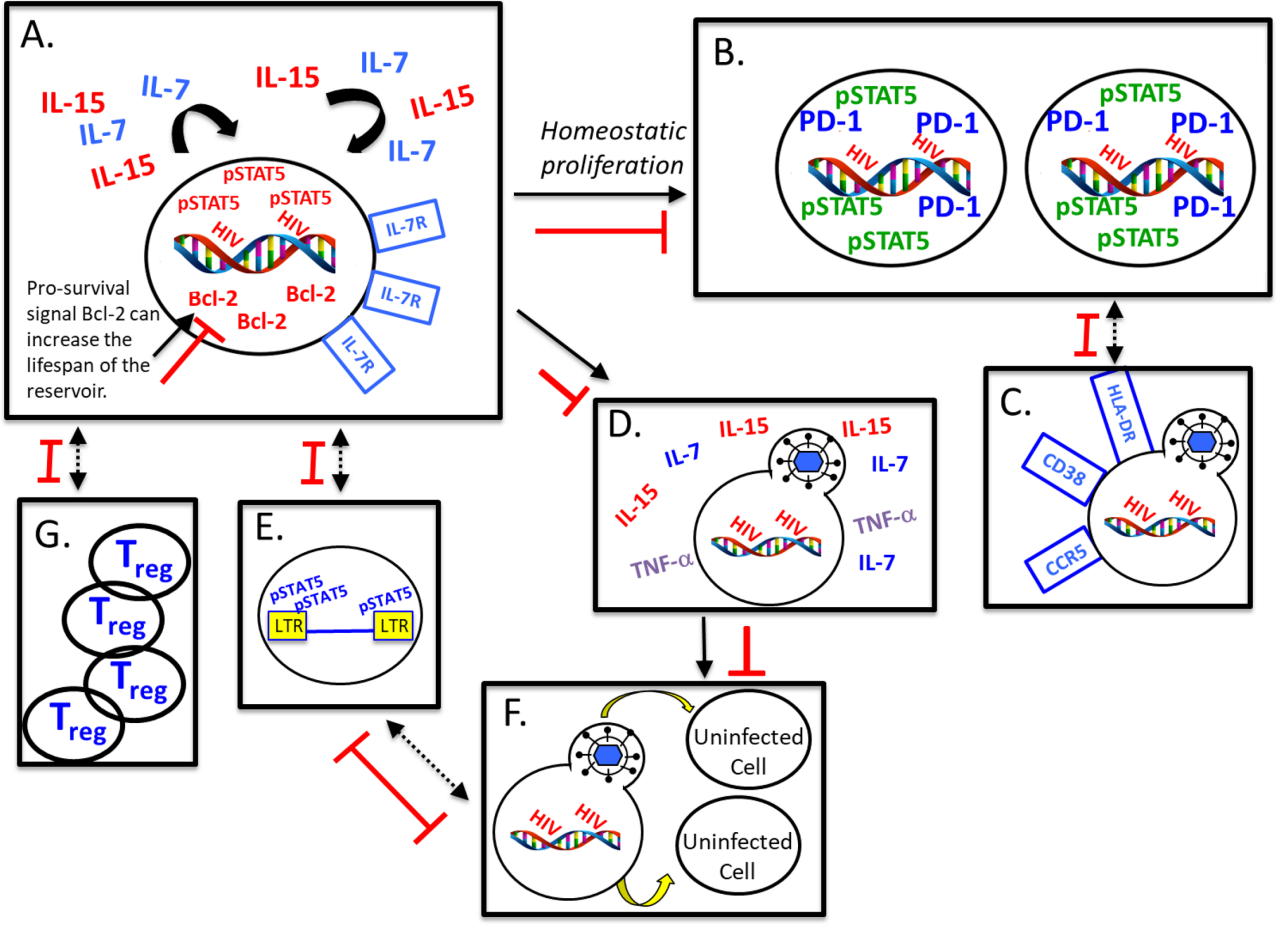
Immunologic mechanisms of viral persistence and impact of Jak inhibitors on the viral reservoir. Positive correlation between pSTAT5 and negative correlation with IL-7R (A) is associated with increased levels of integrated viral DNA (B). PD-1 is associated with levels of integrated viral DNA and homeostatic proliferation (B). T cell activation promotes productive viral replication and increases viral co-receptor, HLA-DR, and CD38, as well as increase in proliferation (C). Pro survival signal Bcl-2 promotes survival of the viral reservoir (A), and IL-15, IL-7, and TNF-α induce reactivation of latent HIV-1, thereby reseeding the viral reservoir (D). HIV LTR shows multiple binding sites for pSTAT5 (E), demonstrating that binding of this transcription factor to the LTR could promote pro-HIV transcripts. Bystander infection in activated cells promotes priming uninfected cells for infection, recruitment of uninfected cells to the site of infection, and reseeding of reservoirs with (D, E, F). STAT5-driven homeostatic proliferation could increase absolute numbers of T_regs_ and lead to further immune dysregulation (G). Red bars indicate pro-HIV events that are blocked by Jak inhibitors.

The IL-7 and IL-15 γ-C cytokines are essential for long term maintenance of memory T cells by regulating homeostatic proliferation and enhancing cell survival which paradoxically leads to HIV persistence (Fig 7A and 7B) [13–15, 17, 18, 20]. Herein, we demonstrate that engagement of this pathway as shown by increased STAT5 phosphorylation is positively associated to the frequencies of cells with integrated HIV DNA and to immune activation which has also been shown to be positively associated with the size of the HIV reservoir. Conversely, we show an inverse relationship between IL-7R levels and integrated HIV DNA in CD4 T cells from ART-treated HIV-infected donors (Table 1; Fig 7A and 7B), confirming the role attributed to STAT5 signaling, in increasing the size of the HIV reservoir by promoting the survival of infected cells expressing receptors for these homeostatic cytokines and by triggering the homeostatic maintenance of these cells (Fig 7A and 7B). Importantly, we show that ruxolitinib or tofacitinib inhibit signaling of the Jak-STAT pathway in all memory T cell subsets known to harbor the HIV reservoir. Our results show that Jak inhibitors impede T cell activation and proliferation both critical for HIV replication and seeding of the reservoir without impeding effector CD4 and CD8 HIV specific responses (Fig 7B–7D). Indeed we show that *ex vivo* and *in vitro*, exposure of T cells to Jak-STAT inhibitors prevents CD3/28-induced up-regulation of several cell surface markers (PD-1, HLADR/CD38 and CCR5) that are expressed by cells that harbor integrated DNA (Table 1, S8–S12 Figs; Fig 7C) [36, 37]. Although Jak inhibitors are not specific to the STAT5 pathway (S2 Fig), careful design of these experiments in the context of cytokines that signal almost exclusively through STAT5, allowed us to assess how blockade of STAT5 contributes to sentinel events driving reservoir establishment, maintenance, and expansion. We also demonstrated that ruxolitinib blocks IFN-α, induced pSTAT1 (CD4^+^ T cells and CD14^+^ monocytes) and IL-10 induced pSTAT3 (CD4^+^ T cells and CD14^+^ monocytes), underscoring that phosphorylation of other STATs may also confer the observed outcome, in combination with blockade of STAT5 (S2 Fig). The involvement of the STAT pathway was confirmed by the findings that exogenous IL-7 could overcome the Jak-inhibitor mediated block on latency reversal, and that Jak inhibitors block IL-15 induced viral reactivation. Importantly, these *ex vivo* and *in vitro* effects were obtained at concentrations (Jakafi.com; [31, 32]) that are achieved during steady-state of this drug in humans for all FDA approved doses. Of note, we previously reported that no apparent toxicity was observed for ruxolitinib or tofacitinib up to 50 μM in T cells and macrophages [34]. Furthermore, decreased viability was primarily observed after the cells from HIV infected subjects were treated with anti-CD3/28 which may be a consequence of HIV infected subjects having lower levels of Bcl-2 and consequently impaired cell survival (20, 44–45).

Our results show that ruxolitinib and tofacitinib can exert direct antiviral potency in infected cells isolated from viremic individuals and in primary CD4 T cells infected *in vitro* (Fig 2). These Jak inhibitors could confer a direct antiviral activity by blocking the phosphorylation of STATs and subsequent binding of this transcription factor to several STAT5 binding sites within the HIV-1 LTR (Fig 7E) [7, 22, 27]. Decreased virus production (summarized in Fig 7C–7F) could also be a consequence of the Jak-STAT induced down-regulation of T cell proliferaton. CD4 T cells exposed to these inhibitors should show decreased expression of transcription factors (NFAT-c, NF-κ-B) expressed by activated T cells [47] and which are required for HIV LTR mediated transcription [48]. Herein, we report that Jak inhibitors demonstrate nanomolar inhibition of viral replication in *ex vivo* CD4 T cells from viremic individuals, even in the presence of an EC_99_ of ART (Fig 2). Therefore, Jak inhibitors may block viral replication in ART treated subjects [33] which highlights their potential as a therapeutic modality, since they could block potential residual viral replication in pharmacological sanctuary sites *in vivo* where antiviral agents do not reach optimal concentrations [45]. Additionally, our results suggest that preferential cell death or shortened lifespan (down regulation of Bcl-2 across T cell subsets; S1 Fig) conferred by Jak inhibitor exposure lead to a reduction in the overall size of the viral reservoir. Dissemination of HIV to bystander cells is also inhibited by addition of Jak inhibitors further confirming the capacity of this class of molecules to prevent further seeding of the reservoir.

CD4 T regulatory cells (Tregs) are the major rheostat of immune homeostasis [49], in addition to suppressing cell mediated immunity to viral infections including HIV infection [50–52]. These cells are dependent on IL-2 and Jak-STAT signaling for their persistence [53]. STAT5-driven homeostatic proliferation is critical for the maintenance of absolute numbers of Tregs [53, 54] where increased number of Tregs could lead to further dysregulation of immune homeostasis (Fig 7, panel G). Jak inhibitors would potentially prevent the increase in Treg homeostatic proliferation in HIV infected subjects which was demonstrated in recent studies of subjects treated with these Jak Inhibitors for myeloproliferative disorders [55]. Inhibition of Treg function would trigger potent cell mediated immune responses that could control residual viral replication [5, 56, 57]. Indeed, ruxolitinib inhibits the production of IL-6, TNF-α IFN-α/β, D-dimer, IL-10, and IL-1α/β *in vivo* [32], markers associated with immune senescence and maintenance of the HIV reservoir. Together, these findings demonstrate that the anti-inflammatory properties of ruxolitinib that are associated with reduced activation and proliferation of T cells in an HIV-infected host could result in reduced bystander cell infection (Fig 7F), diminished levels of homeostatic proliferation (Fig 7B), and decreased permissiveness of uninfected cells by down-regulating the CCR5 co-receptor (Fig 7C).

Lastly, our results also show that this class of compounds can impact HIV reactivation without affecting TCR signaling and function. Indeed, we show that the most proximal events of TCR signaling as well as TCR induced effector function (*i*.*e*. cytokine production) are not affected by Jak inhibitors, although these molecules inhibit TCR induced proliferation. This is in line with previous observations showing that the threshold of MHC/peptide complexes required to trigger antigen specific effector functions is different than that required to induce T cell proliferation. Huang *et*. *al*. showed that a single peptide-major histocompatibility complex ligand interaction with a CD4^+^ T cell is enough to stimulate cytokine production in CD4^+^ T cells [58]. Weak signaling of the TCR still allows for phosphorylation of the TCR signaling pathway and production of cytokines, however only optimal TCR signaling may allow for T cell differentiation and proliferation [46, 58, 59]. This is consistent with our data, wherein steady-state plasma concentrations found *in vivo* (defined as physiological concentrations) did not impair TCR signaling or cytokine production while at the same time decreased CD4 T cell proliferation. Importantly, ruxolitinib did not block HIV gag-peptide T cell responses in CD4 T cells and only decreased CD8 response in 2 of 3 subjects at concentrations ≥ 0.1 μM, indicating that ruxolitinib will not negatively impact HIV specific responses in CD4 T-cells.

Jak inhibitors represent a potential therapeutic modality that addresses a clinical need which traditional direct-acting antiviral agents that interfere with steps in the viral replication cycle have not been successful. We demonstrate that Jak inhibitors confer STAT5-mediated block in homeostatic proliferation, and inflammation-driven HIV reservoir expansion. The effects of Jak inhibitors, described herein on mechanisms that promote HIV dissemination and persistence may be advantageous as an alternative therapeutic intervention that could be implemented in future curative strategies. The impact of ruxolitinib on inflammation associated with HIV infection, which plays an important role in seeding viral reservoirs, viral persistence and ongoing low-level replication in sanctuary sites is currently being evaluated in an NIH-ACTG sponsored 21-site Phase 2a study in humans that is currently enrolling.

## Materials and methods

### Ethics statement

Written informed consent was provided to study participants and approved by the Vaccine and Gene Therapy Institute of Florida ethics review board. Research conformed to ethical guidelines established by the ethics committee of the Vaccine and Gene Therapy Institute of Florida. IRB consent is encompassed under IRB # IRB00006031, obtained on April 5, 2010 for all subjects included in this study.

### Viral load quantification from patient plasma

All multivariate (Table 1) and univariate analyses (S2 Table) were performed with ART treated aviremic donors (S1 Table). To confirm aviremic status, plasma viral load was measured with the Amplicor HIV Monitor Ultrasensitive Method (Roche, Basel, Switzerland), wherein donors were considered aviremic when viral load was below 50 copies/ml.

### Isolation of CD4 T lymphocytes

Human peripheral blood mononuclear cells (PBMCs) were isolated from leukapheresis by density gradient centrifugation as previously described [13]. Total and memory CD4^+^ T cells were isolated from PBMC of viremic, aviremic or HIV-negative individuals using magnetic bead–based negative selection (Stemcell Technologies, Vancouver, British Columbia, Canada). Cells were used for antiviral potency, signaling, or reservoir studies as described below.

### Quantification of HIV production and reactivation

A previously described, in-house p24 ELISA [60] was used to quantify viral production by CD4^+^ cells isolated from viremic donors *ex vivo* and *in vitro* infected CD4^+^ cells cultured in the presence of Jak inhibitors. Briefly, freshly enriched CD4^+^ T cells from viremic donors (1 to 1.5 x 10^6^ cells) were stimulated for 3–6 days with 1 μg/mL of anti-CD3 (OKT3 hybridoma cell line; NIH AIDS and reference reagent program) and 1 μg/mL of anti-CD28 (BD Biosciences, Franklin Lakes, New Jersey) in the presence of increasing concentrations of Jak inhibitors either with or without ART (180 nM zidovudine, 100 nM efavirenz, 200 nM raltegravir (Cat # 11680) from Merck & Company, Inc.; NIH AIDS and reference reagent program, Division of AIDS, NIAID, NIH). Enriched CD4^+^ T cells isolated from HIV negative donors were pre-activated for 3 days with anti-CD3/CD28, infected with an eGFP NL4-3 replication competent HIV-1 reporter virus (pBR43IeG-nef^+^ (Cat #11349) from Dr. Frank Kirchhoff, NIH AIDS and reference reagent program, Division of AIDS, NIAID, NIH) [61–63] and cultured for 3–6 days in the presence of increasing concentrations of Jak inhibitors. Serial dilutions of cell-free supernatant from the ACH2 cell line with a known p24 concentration were used for the standard curve.

To determine if Jak inhibitors block IL-15-induced reactivation, 5 x 10^6^ CD4^+^ T cells from aviremic donors were isolated and cultured with 1.0 μM ruxolitinib (Selleck Chemicals, Boston, MA, USA) or DMSO and either 10 ng/ml IL-15 or CD3/CD28 for 6 days in the presence of ART (180 nM zidovudine, 100 nM efavirenz, 200 nM raltegravir). Viral quantification (HIV particles containing HIV RNA) in cell-free supernatants was measured by qRT-PCR as previously described [14] and compared to an unstimulation control. Experiments were conducted in three independent donors.

To establish IL-7 rescue of virus reactivation from Jak inhibition, freshly isolated CD4^+^ T cells from viremic donors (1.5 x 10^6^ cells) were pre-incubated with anti-CD3/CD28 and 0.033 μM ruxolitinib for 30 min prior to addition of 30 ng/mL IL-7. Quantification of virus in supernatants from these cultures was measured by p24 ELISA after a 6-day culture. Experiments were conducted in four independent donors.

### Quantification of integrated HIV DNA

To quantify the frequency of cells harboring integrated viral DNA, fresh CD4^+^ T cells isolated from viremic donors were cultured in the presence of Jak inhibitors and stimulated with anti-CD3/CD8 with or without ART (180 nM zidovudine, 100 nM efavirenz, 200 nM raltegravir) and lysed at the termination of the experiment on Day 6. Cell lysates were directly used in a nested Alu PCR. This method provides an accurate quantification of the absolute numbers of cells per million that carry integrated HIV DNA, as previously described [13, 14].

### Measurement of cell activation / proliferation, HIV co-receptors and Bcl-2 expression

The expression of proliferation/activation markers and HIV co-receptors were monitored by flow cytometry using the following antibody panel: CD3-A700 (clone UCHT1, BD Biosciences, Franklin Lakes, New Jersey), CD4-Qdot605 (clone S3.5, Invitrogen, Carlsbad, California), CD8–PerCP Cy5.5 (clone RPA-T8, eBiosciences, San Diego, CA), HLA-DR–PerCP (clone L243, BD Biosciences), CD38-PE (clone HIT2, BD Biosciences), CD25-PE-Cy7 (clone M-A251, BD Biosciences) or PD-1-PE-Cy7 (clone EH12.2H7, BioLegend, San Diego, CA), AnnexinV-APC (BD Biosciences) and Cell Trace Violet (CTV; which was applied at the beginning of culture to allow to track proliferation due to dilution of the dye; Invitrogen). CCR5 and CXCR4 expression was evaluated using the panel described above replacing AnnexinV-APC and CD38-PE with CXCR4-APC (clone, 12G5, eBioscience) and CCR5-PE (clone 2D7, BD Biosciences). Bcl-2 was quantified using anti-Bcl-2-Pacific Blue (clone Bcl-2/100, BD Biosciences) in combination with CD3-A700, CD4-Qdot605, CD8-PerCP (clone SK1, BD Biosciences), CD45RA-BV650 (clone HI100, BioLegend), CCR7-FITC (clone 150503, R&D Systems) and CD27-APCeFluor780 (clone O323, eBiosciences). For all stains, dead cells were excluded with the LIVE/DEAD Aqua marker (Invitrogen). For all flow cytometry panels, cells were acquired on an LSRII flow cytometer using the FACSDiva software (Becton Dickinson, Franklin Lakes, New Jersey) and analyzed using FlowJo v9.9.6 and v10.2 (TreeStar Inc., Ashland, Oregon).

### Measurement of Jak-STAT and T cell receptor signaling by PhosFlow

To measure the impact of Jak inhibitors on γ-C-cytokine-mediated STAT5 phosphorylation, PBMCs were first pre-incubated for 60 minutes with ruxolitinib (Selleck Chemicals, Boston, MA, USA) or tofacitinib (Selleck Chemicals, Boston, MA, USA) followed by a LIVE/DEAD stain. Stained cells were washed and incubated at 37°C with 50 ng/mL IL-2 (R&D Systems, Minneapolis, MN), 2 ng/mL IL-7 (R&D Systems), 5 ng/mL IL-15 (R&D Systems), 10 ng/mL IL-10 (R&D Systems) or 10,000 Units IFN-α A (R&D Systems) in the presence of compound. After 15 minutes, cells were fixed with BD Cytofix Fixation Buffer (BD Biosciences) and permeabilized with Perm Buffer III (BD Biosciences). Permeabilized cells were stained with CD3-A700, CD4-Qdot605, CD8-Pacific Blue, CD45RA-BV650, CCR7-FITC, CD27-APCeFluor780, STAT1 (pY701)-A647 (clone 4a, BD Biosciences), STAT3 (pY705)-PE (clone 4/P-STAT3, BD Biosciences) and STAT5 (pY694)-PE-CF594 (clone 47/STAT5 (pY694), BD Biosciences).

To measure the impact of Jak inhibitors on T cell receptor (TCR) signaling, bead enriched CD4^+^ T cells were pre-incubated with ruxolitinib for 30 minutes followed by a cell surface stain with LIVE/DEAD dye in the presence of compound to exclude dead cells. Stained cells were washed and cross-linked in the presence of compound for 10 minutes on ice with 0.5 μg/mL anti-CD3 (OKT3), 5 μg/mL anti-CD28 (BioLegend) and 10 μg/mL goat anti-mouse IgG (BioLegend). After 10 min, cells were fixed with BD Cytofix Fixation Buffer (BD Biosciences) and permeabilized with Perm Buffer III (BD Biosciences). Permeabilized cells were stained with CD3-A700, CD4-Qdot605, CD3 Zeta (pY142)-A647 (clone K25-407.69, BD Biosciences) and SLP76 (pY128)-PE (clone J141-668.36.58, BD Biosciences).

### Measurement of cytokine production by intracellular staining

To measure the impact of Jak inhibitors on TCR-induced cytokine production in the absence of HIV infection, PBMCs were isolated from HIV negative donors, pre-treated for 1 hr with 0.01–10 μM ruxolitinib and stimulated for 6 hours with anti-CD3/CD28 in the presence of Brefeldin A (5μg/ml) and increasing concentrations of ruxolitinib *versus* DMSO. Following stimulation, cells were stained with CD3-PB, CD4-APC, CD8-PerCP and permeabilized with saponin. Permeabilized cells were then stained with fluorochrome-conjugated mAbs to the following cytokines: IL-2 FITC (clone MQ1-17H12, BD Biosciences), TNF-α A700 (clone MAb11, BD Biosciences), and IFN-γ PE-Cy7 (clone 4S.B3, BD Biosciences).

To measure the impact of ruxolitinib on CD4 and CD8 T cell responses to HIV gag peptide, total PBMCs from ART-suppressed HIV^+^ donors were thawed and rested overnight in media containing antiretrovirals to a final concentration of 200 nM raltegravir, 100 nM efavirenz and 180 nM AZT. The following day, 1.5 million cells per condition were pre-treated as follows: DMSO alone, or with Ruxolitinib at: 0.01 μM, 0.1 μM, 1.0 μM, or 10 μM for one hour at 37°C. Following the one hour preincubation, cells were either not stimulated (DMSO alone at a quantity equivalent to that used for gag peptide pool) or stimulated with: 1.0 μg/ml pooled Gag consensus A peptides (Cat #8116 HIV-1 Consensus A Gag Peptide Set, NIH AIDS Reagent Program) or platebound 1.0 μg/ml OKT3 anti-CD3 (hybridoma clone). All stimulations included brefeldin A (Sigma, Cat# B5936) at 10 μg/ml, soluble 1.0 μg/ml anti-CD28 (BD Biosciences), and were performed for 6 hours. Ruxolitinib was included at the previously noted concentrations for the full 6 hours. The intracellular cytokine staining assay was performed as follows. Stimulated samples were stained with live/dead fixable violet viability dye (Invitrogen) and the following surface antibody panel: anti-CD19 BV510 (clone HIB19, Biolegend), anti-CD4 Qdot605 (Invitrogen), anti-CD14 V500 (clone M5E2, BD Biosciences), anti-CD8 BV711 (clone RPA-T8, BD Biosciences) and anti-CD3 Alexa700 (BD Biosciences). After a single wash cells were fixed in 2% formaldehyde (paraformaledhyde) for 10 min at room temperature, then permeabilized with 0.05% Saponin. The following antibodies were added for intracellular staining: anti-TNF-α Alexa Fluor 488 (clone MAb11, BD Biosciences) and anti-IFN-γAPC (clone B27, Biolegend). Cells were washed, fixed in 2% PFA, and data was collected on a Fortessa flow cytometer (BD Biosciences) and analyzed using FlowJo software (TreeStar Ashland, OR, USA).

### Bystander cell infection assay

To quantify the impact of ruxolitinib on bystander infection of CD4^+^ T cells by HIV, CD4^+^ T cells isolated from HIV-negative individuals were stained with 0.1 μg/ml Cell Trace Violet (CTV) dye (Invitrogen) to track and quantify bystander cells that become infected (CTV^+^/GFP^+^) or left unstained. Cells with CTV dye were stimulated for 3 days with anti-CD3/CD28 in the presence of various concentrations of ruxolitinib or DMSO (negative control). Cells without CTV were stimulated with anti-CD3/28 for 3 days and spinoculated for two hours at RT (at 2000g) with NL4-3 GFP reporter virus. After two additional hours incubation at 37°C, infected CTV negative cells were washed in RPMI plus 2% Fetal Bovine Serum and co-cultured with uninfected CTV positive cells (ratio 1:1) for an additional two days. Bystander infected cells were identified by the expression of GFP in CTV positive cells.

### Pharmacokinetic (PK) simulation of ruxolitinib plasma concentrations

To confirm that the observed *in vitro* and *ex vivo* anti-HIV effects mediated by ruxolitinib occur within the steady-state concentration range observed in humans, a pharmacokinetic simulation was performed. Ruxolitinib plasma concentrations were simulated for 10 and 20 mg bid dose regimens, using parameters of the basic 2-compartmental population pharmacokinetics model with first-order oral absorption (not including patient covariates), fitted to data from male patients (163 of 272 patients) from Phase 1 and 2 trials undergoing treatment for myelofibrosis. Males are the primary subjects of an ACTG trial (NCT02475655) of ruxolitinib in HIV infected subjects; hence males were utilized for this model [31]. Monte-Carlo simulations were performed to predict concentration *versus* time profiles of 1,000 theoretical subjects, and used to compute percentile ranges (P_10_, P_25_, P_50_, P_75_, and P_90_) of plasma concentrations *versus* time over the first 36 hours after start of ruxolitinib, which were then plotted. Since ruxolitinib is about 90% bound to serum in humans (ruxolitinib package insert), and cells were incubated in media with 10% fetal bovine serum (FBS), the *in vitro* EC_50_ were multiplied by 10 before plotting. The median and % CV of pharmacokinetic parameters used for the simulation were: first order absorption rate constant (K_a_ = 3.43 hr-1, 75%), oral systemic clearance (CL/F = 20.2 L/hr., 37.9%, inter-compartmental clearance (Q/F = 2.6), central and peripheral volumes of distribution (V_c_/F = 57.7 L, 30.9%; V_p_ = 11.8 L, 85.7), absorption lag-time (p.052 hr.) and residual variance (⌠^2^ = 35.8%). Inter-individual variances were modeled as proportional (log-normally distributed), noting that the formula for % CV for a log-normal distributed parameter = ℘(e^⎤^2^–1) x 100, where ⎤^2^ = variance of a log-normally distributed parameter. Simulations were run using the NONMEM program (7.3 ICON Development Solutions, Ellicott City, MD), and statistics and graphical analyses were performed using R 3.0.1 (R Statistical Foundation, Vienna Austria, https://cran.r-project.org/web/packages/RODBC/index.html).

### Association between ex vivo markers of the Jak-STAT pathway and integrated HIV DNA

For identifying markers of the Jak-STAT pathway that are associated with the size of the HIV reservoir, we fit a linear model with frequencies of cells harboring integrated HIV DNA as a dependent variable and the different markers as independent variables (S2 Table). The obtained p-values were then corrected for multiple comparisons using the Benjamini and Hochberg (BH) method. Feature selection was performed using the Least Absolute Shrinkage and Selection Operator (LASSO) technique [39], in order to determine the combination of the significant univariate markers that best predict the size of the HIV reservoir (Table 1). The model was optimized using leave-one-out cross validation and the least cross-validated mean square error (MSE) was determined.

For STAT5 phosphorylation or Bcl2 expression across CD4 memory subsets, a linear regression model was applied with Bcl2 MFI (or %pStat5) as the dependent variable and the drug as the independent variable taking concentration into consideration (S4 Table). For HIV gag-peptide responses in CD4 or CD8 Tcells, a paired Wilcoxon rank sum test was used to determine significance (Fig 5 and S18 Fig).

For quantification of HIV production and reactivation; quantification of integrated HIV DNA; measurement of cell activation, proliferation, HIV co-receptors and Bcl-2 expression; measurement of Jak-STAT and T cell receptor signaling; and measurement of cytokine production following TCR signaling, significance was determined by two-way ANOVA followed by Sidak’s multiple comparison. Statistics were performed using GraphPad Prism 6.0 software. A p-value less than 0.05 was considered statistically significant.

## Supporting information

S1 Fig STAT5 phosphorylation or Bcl2 expression across CD4 memory subsets in the presence of ruxolitinib or tofacitinib. Bcl2 MFI (A, C) or percent STAT5 phosphorylation (pY694) (B, D) in CD4 memory subsets measured by flow cytometry in PBMC from 3 healthy subjects after 15 minute stimulation with 50 ng/mL IL-2, 2 ng/mL IL-7 or 5 ng/mL IL-15 and 0.01, 0.1 and 1 μM ruxolitinib, tofacitinib or DMSO. Data represented as a linear regression with Bcl2 MFI (or %pStat5) as the dependent variable and the drug as the independent variable taking concentration into consideration. Jak inhibitors significantly (p < 0.05) reduced Bcl-2 expression (MFI) and % of pSTAT5^+^ cells. CM–central memory; TM–transitional memory and EM–effector memory.
(PDF)

S2 Fig Jak inhibitors block cytokine-induced STAT1, STAT3 and STAT5 phosphorylation. STAT1, STAT3 or STAT5 phosphorylation (% in CD4+ T cells and CD14+ monocytes) was measured by flow cytometry in PBMC isolated from HIV negative donors and stimulated for 15 min with 10,000 Units IFN-α (A-D) or 10 ng/ml IL-10 (E-F) (n = 3) and increasing concentrations (0.01, 0.1, and 1.0 μM) of ruxolitinib. 0.0 μM represents the average of all assays completed using % DMSO equivalent to Jak inhibitor concentrations. Error bars represent standard deviation and statistical significance determined by two-way ANOVA followed by Sidak’s multiple comparison post-test: *p < 0.05, **p < 0.01, ***p < 0.001 and ****p < 0.0001.
(PDF)

S3 Fig Jak inhibitors block HIV-1 replication *ex vivo*. Raw data of viral replication measured by ELISA p24 in cell-free supernatants of enriched CD4^+^ T cells isolated from 5 viremic donors and stimulated for 6 days with anti-CD3/28 in the presence of increasing concentrations of Jak inhibitors without ART.
(PDF)

S4 Fig Jak inhibitors block HIV-1 production *ex vivo*. Raw data of viral production measured by ELISA p24 in cell-free supernatants of enriched CD4^+^ T cells isolated from 5 viremic donors and stimulated for 6 days with anti-CD3/28 in the presence of increasing concentrations of Jak inhibitors with ART.
(PDF)

S5 Fig Jak inhibitors inhibit anti-CD3/28 upregulation of HIV co-receptor CCR5 in viremic donors. HIV coreceptor CCR5 was quantified in CD4^+^ T cells isolated from viremic donors and cultured for 6 days as in (Fig 2A and 2B). Percentage of CD4 cells expressing CCR5 from individual donors (A). To account for inter-patient variability in baseline values, results in B are reported as the fold change *versus* DMSO controls. 0.0 μM represents the average of all assays completed using % DMSO equivalent to Jak inhibitor concentrations. Error bars represent S.E.M. and statistical significance determined by two-way ANOVA followed by Sidak’s multiple comparison post-test: *p < 0.05, **p < 0.01, ***p < 0.001 and ****p < 0.0001.
(PDF)

S6 Fig Jak inhibitors do not change HIV co-receptor CXCR4 expression in viremic donors. HIV coreceptor CXCR4 was quantified in CD4^+^ T cells isolated from viremic donors and cultured for 6 days as in (Fig 2A and 2B). Percentage of CD4 cells expressing CXCR4 from individual donors (A). To account for inter-patient variability in baseline values, results in B are reported as the fold change *versus* DMSO controls. 0.0 μM represents the average of all assays completed using % DMSO equivalent to Jak inhibitor concentrations. Error bars represent S.E.M. and statistical significance determined by two-way ANOVA followed by Sidak’s multiple comparison post-test: *p < 0.05, **p < 0.01, ***p < 0.001 and ****p < 0.0001.
(PDF)

S7 Fig Reversal of ruxolitinib-mediated inhibition of viral replication by exogenous addition of IL-7. CD4 T cells from viremic donors (n = 4) were pre-incubated with anti-CD3/CD28 and 33 nM Ruxolitinib 30 min prior to addition of IL-7 (30 ng/mL). p24 was measured after 6 days in culture. Error bars represent S.E.M. and statistical significance determined by paired T-test (A), where DMSO controls without cytokine versus DMSO control + IL-7 was compared (paired t-test) and Ruxolitinib (no cytokine) was compared to ruxolitinib (+ IL-7) (paired t-test). * p < 0.05 compared to no cytokine addition. p24 measurements from each individual donor (B).
(PDF)

S8 Fig Ruxolitinib and tofacitinib inhibit T-cell activation and proliferation in CD4^+^ T cells of viremic donors. Cell proliferation (A) and activation (B-D) as measured by flow cytometry in enriched CD4^+^ T cells isolated from viremic donors and cultured for 6 days with CD3/28 and increasing concentrations of Jak inhibitors in the absence of antiretroviral agents [**(-);** designed to observe the effect of ruxolitinib alone, in the presence of ongoing replication] or presence of 180 nM zidovudine, 100 nM efavirenz, 200 nM raltegravir [**(+);** to observe the effect of ruxolitinib when all spreading infection is inhibited] (n = 5). Percentage of cells expressing CD25 (B), HLA-DR/CD38 (C), PD-1 (D) and low levels of Cell Trace Violet [CTV] (A). To account for inter-patient variability in baseline values, results are reported as the fold change *versus* DMSO treated control cells. Activation and proliferation markers by the latter are normalized to 1. Error bars represent S.E.M. and statistical significance determined by two-way ANOVA followed by Sidak’s multiple comparison post-test: * p < 0.05, ** p < 0.01, *** p < 0.001 and **** p < 0.0001.
(PDF)

S9 Fig Ruxolitinib and tofacitinib inhibit proliferation in CD4^+^ T cells of viremic donors. Cell proliferation as measured in S8 Fig in individual donors.
(PDF)

S10 Fig Ruxolitinib and tofacitinib inhibit CD25 expression in CD4^+^ T cells of viremic donors. CD25 expression as measured in S8 Fig in individual donors.
(PDF)

S11 Fig Ruxolitinib and tofacitinib inhibit CD38/HLA-DR expression in CD4^+^ T cells of viremic donors. CD38/HLA-DR expression as measured in S8 Fig in individual donors.
(PDF)

S12 Fig Ruxolitinib and tofacitinib inhibit PD-1 expression in CD4^+^ T cells of viremic donors. PD-1 expression as measured in S8 Fig in individual donors.
(PDF)

S13 Fig Representative gating strategy for TCR signaling. (PDF)

S14 Fig **Gating strategy (A) and impact of ruxolitinib on (B) TNF-α+, (C) IFN-γ+ or (D) IL-2+ expressing CD4 or CD8 cells.** Mean cytokine production (% of IL-2^+^, TNF-α^+^ or IFN-γ^+^ positive cells) in CD3+CD8- cells or CD3+CD8+ cells as measured by flow cytometry in PBMC isolated from HIV negative donors and stimulated for 6 hr with aCD3/CD28, Brefeldin A (5 μg/ml) and increasing concentrations of Ruxoltinib *versus* DMSO treated cells (n = 3). 0.0 μM represents the average of all assays completed using % DMSO equivalent to Jak inhibitor concentrations. Statistical significance determined by two-way ANOVA followed by Sidak’s multiple comparison post-test: *p < 0.05, **p < 0.01, ***p < 0.001 and ****p < 0.0001.
(PDF)

S15 Fig Jak inhibitors impair cell viability in cells from HIV infected viremic donors. The frequency of live cells (AnnexinV- and Live/Dead viability mearker -) as measured by flow cytometry in enriched CD4^+^ T cells isolated from viremic donors and cultured for 6 days with CD3/28 and increasing concentrations of Jak inhibitors in the absence of antiretroviral agents [**(ART-);** designed to observe the effect of ruxolitinib or tofacitinib alone, in the presence of ongoing replication] or presence of 180 nM zidovudine, 100 nM efavirenz, 200 nM raltegravir [**(ART+);** to observe the effect of ruxolitinib or tofacitinib when all spreading infection is inhibited] (n = 5) (A). The frequency of live cells (AnnexinV- and Live/Dead viability marker -) measured after 3 day culture of *in vitro* infection of CD4^+^ cells from 3 healthy donros in the presence of increasing concentrations of Jak inhibitors with ART as described in Fig 2D (B). Frequency of live cells (Live/Dead viability marker -) measured after 6 day culture of CD4^+^ cells from healthy donors in the presence of increasing concentrations of Jak inhibitors and IL-2, -7 or -15 as described in Fig 1C.
(PDF)

S16 Fig Ruxolitinib and tofacitinib inhibit activation of *in vitro* infected CD4 T cells. Activation (A-C) and proliferation (D) measured by flow cytometry of *in vitro* infected CD4^+^ T cells after 3 days culture with anti-CD3/28 in the presence of increasing concentrations of Jak inhibitors and ART (180 nM zidovudine, 100 nM efavirenz, 200 nM raltegravir) [N = 3] as described in Fig 2D. Percentage of cells expressing HLA-DR (A), CD38 (B), CD25 (C) and low levels of Cell Trace Violet [CTV] (D).
(PDF)

S17 Fig Distribution of STAT5 phosphorylation in aviremic donors classified by CD4 count. pSTAT5 MFI between non immune responders (NIR), Immune Responders (IR), Successfully Treated Non Classified (ST NC) and Recently Treated (RT) was determined in Naïve, CM, TM and EM CD4 T cell subsets by the Wiloxon rank test. A p-value < 0.05 was considered statistically significant.
(PDF)

S18 Fig **Gating strategy (A) and impact of ruxolitinib on (B) TNF-α+ or (C) IFN-γ+ expressing CD4 or CD8 cells.** Mean percent TNF-α^+^ (n = 6) or IFN-γ^+^ (n = 5) cells in CD3+CD4+ cells or CD3+CD8+ cells (n = 3) as measured by flow cytometry in PBMC isolated from stably treated, HIV positive donors and stimulated for 6 hr with 1 μg/ml gag-peptide, Brefeldin A (5 μg/ml) and increasing concentrations of Ruxoltinib *versus* DMSO treated cells. 0.0 μM represents the average of all assays completed using % DMSO equivalent to Jak inhibitor concentrations. Statistical significance determined by paired Wilcoxon rank sum test.
(PDF)

S1 Table Profile of ART-treated HIV infected subjects including CD4, CD8 T cell counts, viral load, time individual has been HIV-infected, and whether individual is receiving ART. (PDF)

S2 Table Univariate analysis of *ex vivo* CD4 T-cells, activation markers, proliferation and STAT signaling with integrated HIV DNA. (XLSX)

S3 Table Differences in activation markers, PD-1/IL-7R and STAT phosphorylation between different classes of aviremic subjects. (XLSX)

S4 Table Linear model for inhibition of Bcl2 or pSTAT5 in CD4 central memory (CM), transitional memory (TM) and effector memory (EM) subsets by ruxolitinib or tofacitinib. (XLSX)
