# Supplementary figures and images for: Novel mechanisms to inhibit HIV reservoir seeding using Jak inhibitors
Source: PLoS Pathog. 2017 Dec 21;13(12):e1006740. doi: 10.1371/journal.ppat.1006740 (PMC5739511; doi:10.1371/journal.ppat.1006740)

**A**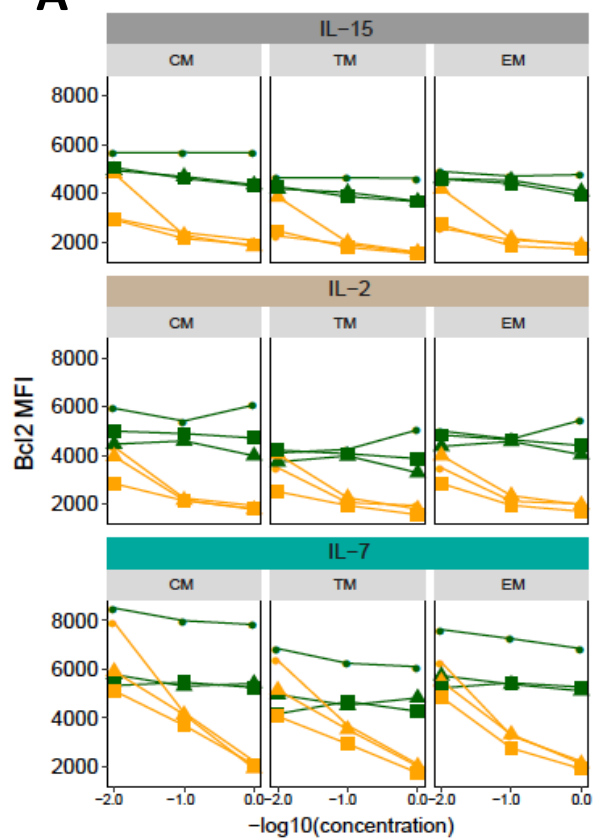**B**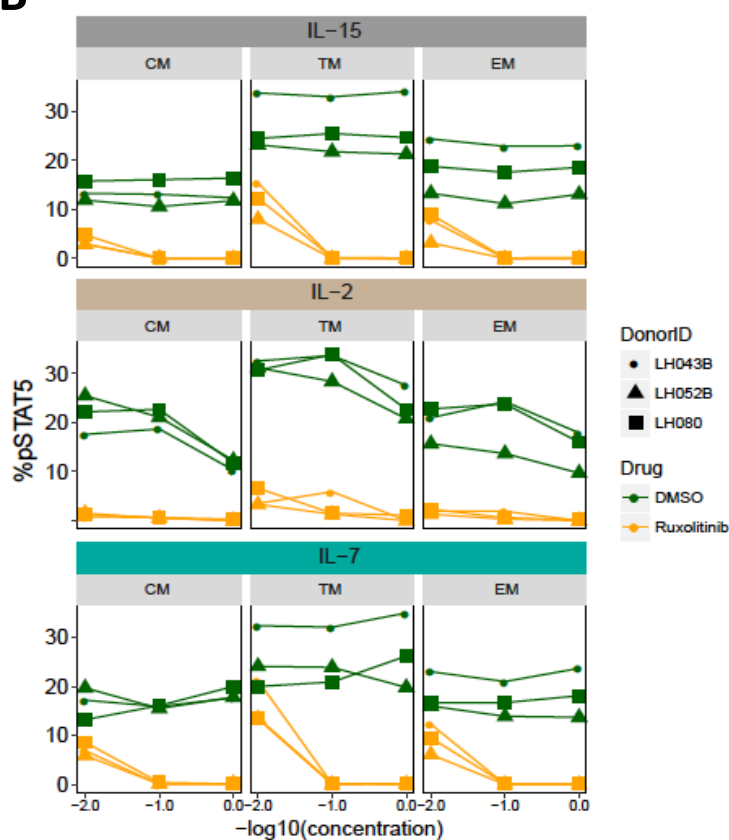**C**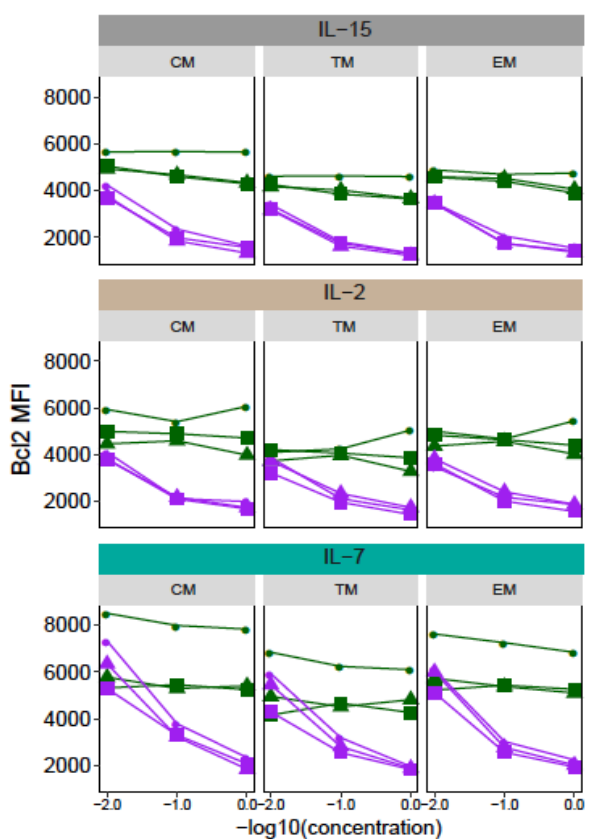**D**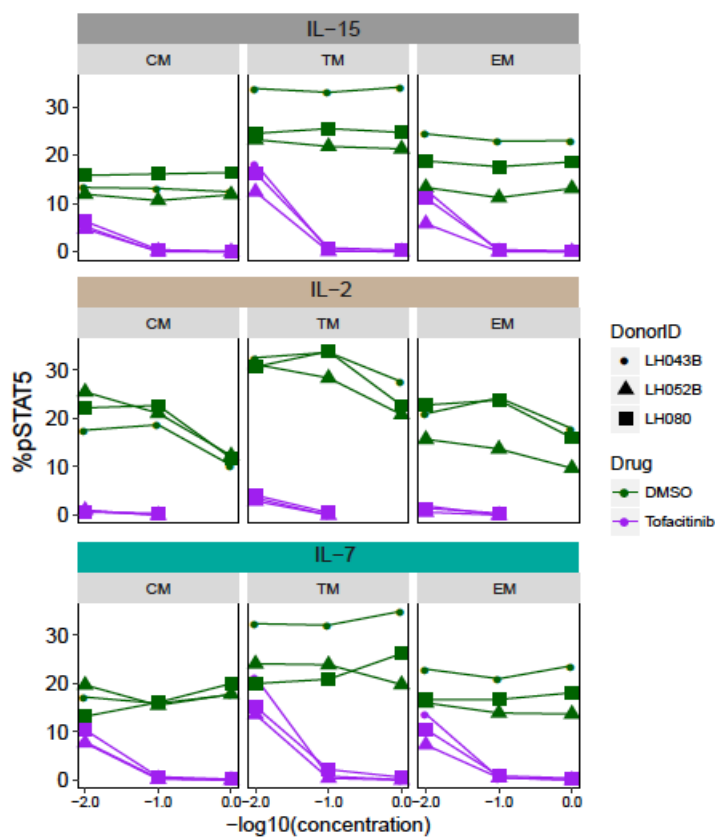

Supplement: S1 Fig — Bcl2 MFI (A, C) or percent STAT5 phosphorylation (pY694) (B, D) in CD4 memory subsets measured by flow cytometry in PBMC from 3 healthy subjects after 15 minute stimulation with 50 ng/mL IL-2, 2 ng/mL IL-7 or 5 ng/mL IL-15 and 0.01, 0.1 and 1 μM ruxolitinib, tofacitinib or DMSO. Data represented as a linear regression with Bcl2 MFI (or %pStat5) as the dependent variable and the drug as the independent variable taking concentration into consideration. Jak inhibitors significantly (p < 0.05) reduced Bcl-2 expression (MFI) and % of pSTAT5+ cells. CM–central memory; TM–transitional memory and EM–effector memory. (PDF) [file ppat.1006740.s001.pdf]

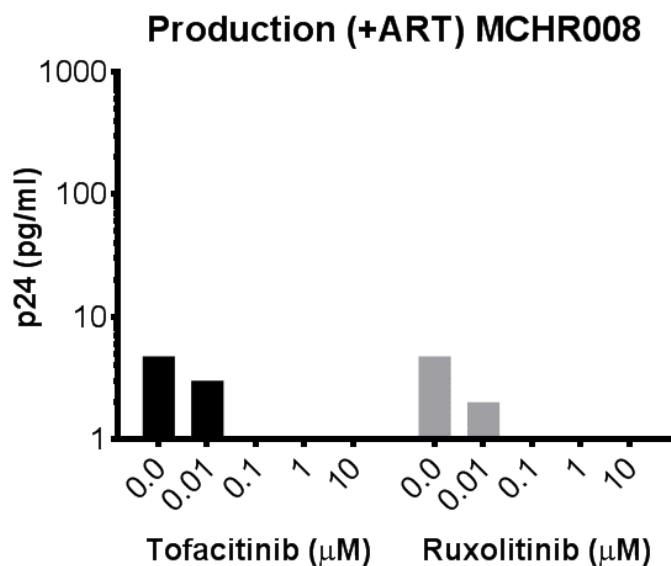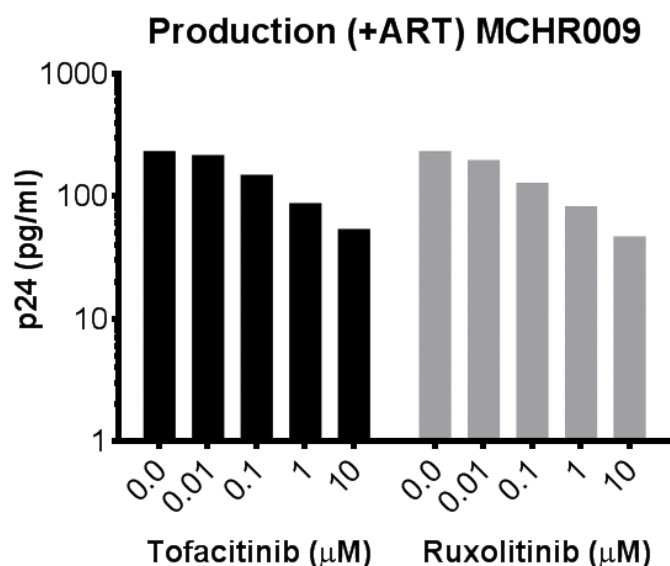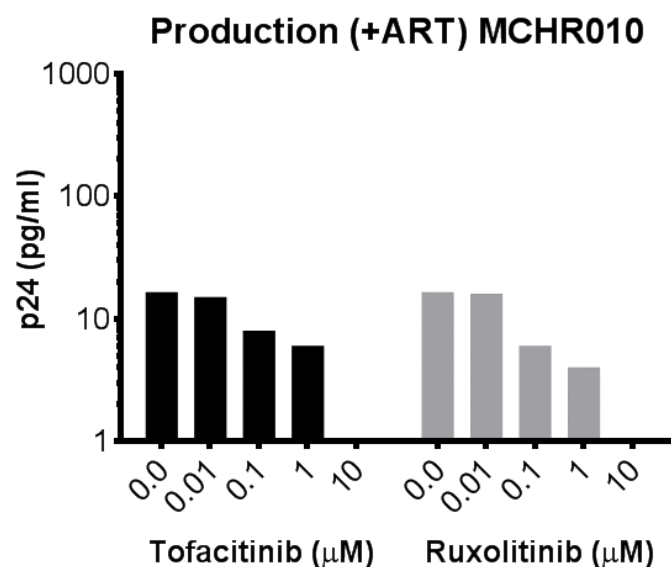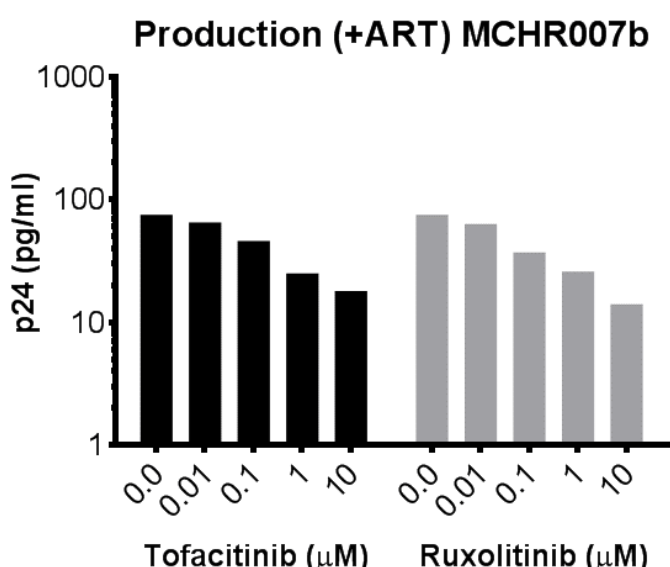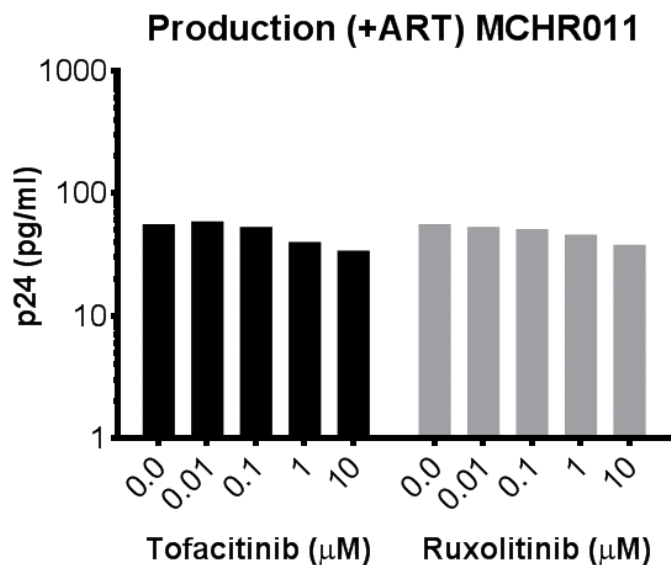

Supplement: S4 Fig — Raw data of viral production measured by ELISA p24 in cell-free supernatants of enriched CD4+ T cells isolated from 5 viremic donors and stimulated for 6 days with anti-CD3/28 in the presence of increasing concentrations of Jak inhibitors with ART. (PDF) [file ppat.1006740.s004.pdf]

**A**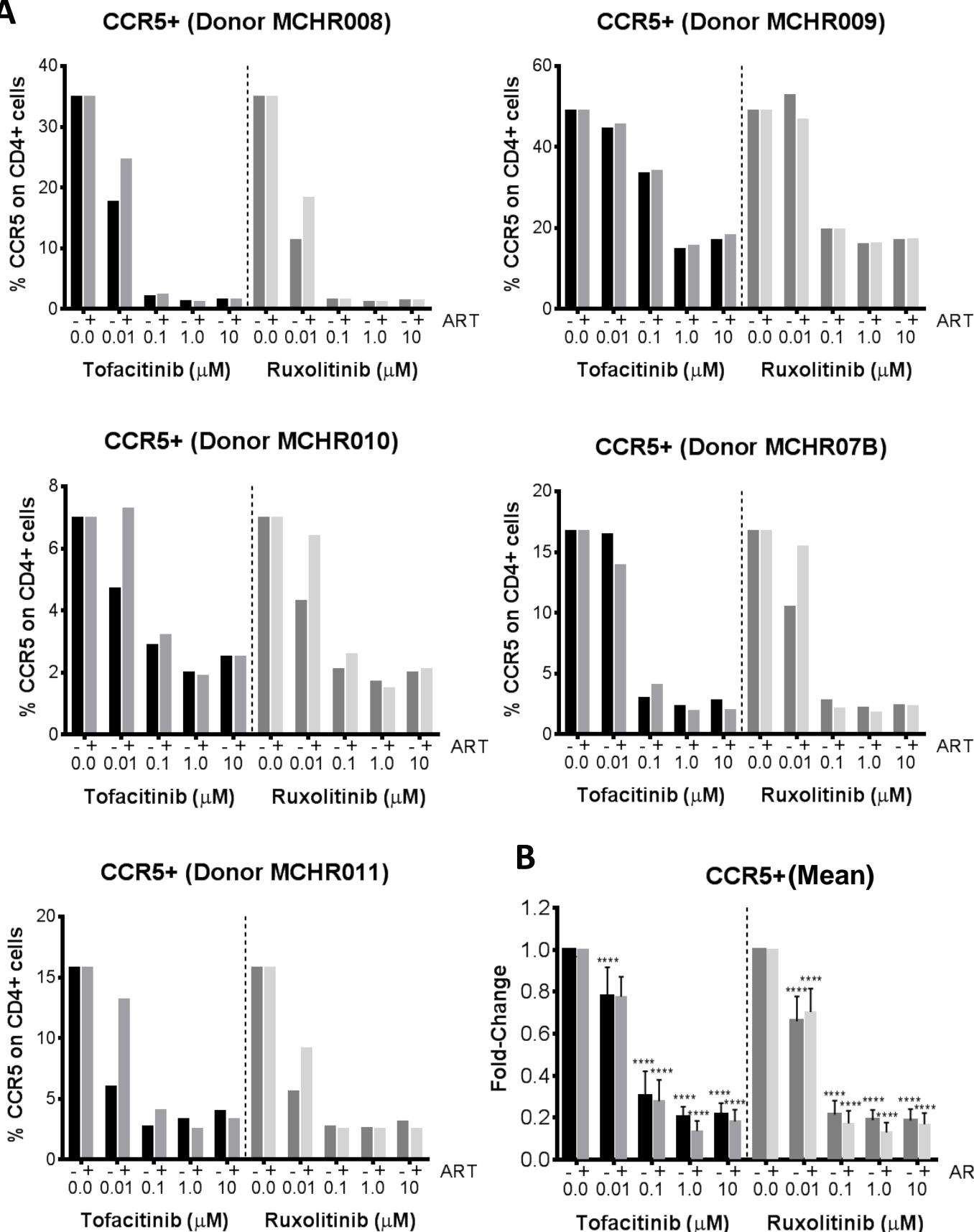

Supplement: S5 Fig — HIV coreceptor CCR5 was quantified in CD4+ T cells isolated from viremic donors and cultured for 6 days as in (Fig 2A and 2B). Percentage of CD4 cells expressing CCR5 from individual donors (A). To account for inter-patient variability in baseline values, results in B are reported as the fold change versus DMSO controls. 0.0 μM represents the average of all assays completed using % DMSO equivalent to Jak inhibitor concentrations. Error bars represent S.E.M. and statistical significance determined by two-way ANOVA followed by Sidak’s multiple comparison post-test: *p < 0.05, **p < 0.01, ***p < 0.001 and ****p < 0.0001. (PDF) [file ppat.1006740.s005.pdf]

**A****CXCR4+ (MCR008)**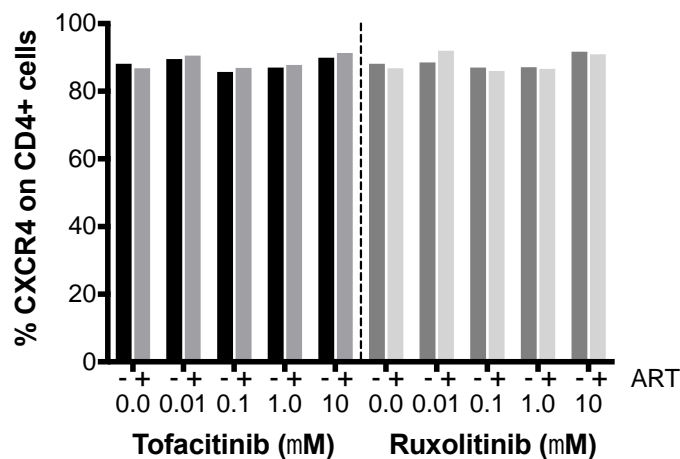**CXCR4+ (MCR009)**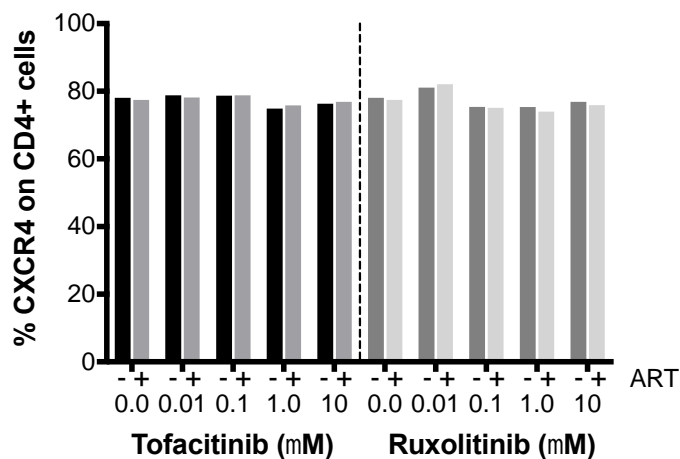**CXCR4+ (MCR010)**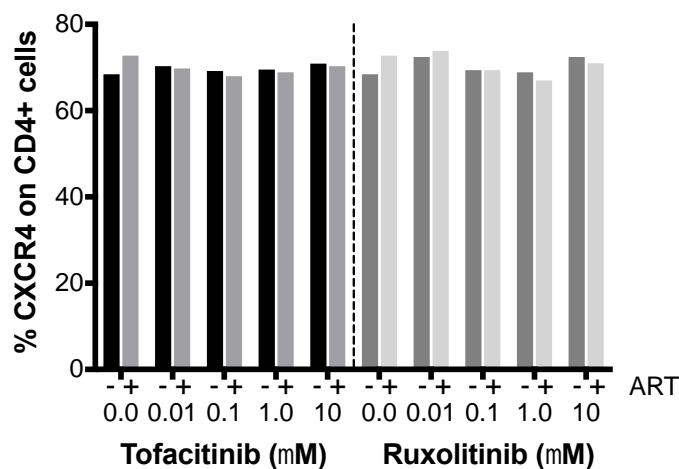**CXCR4+ (MCR007b)**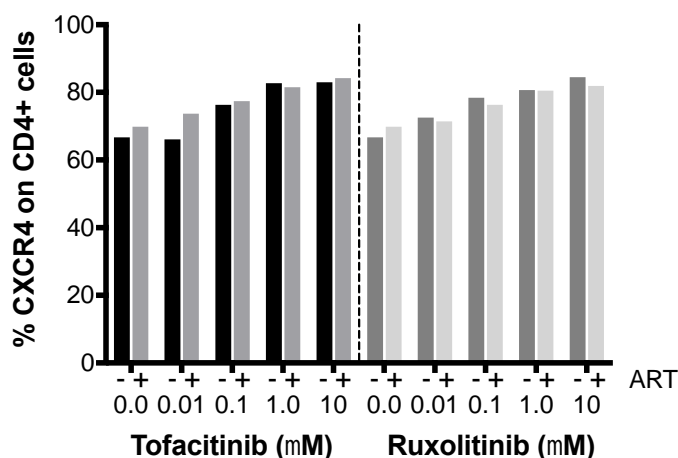**CXCR4+ (MCR011)**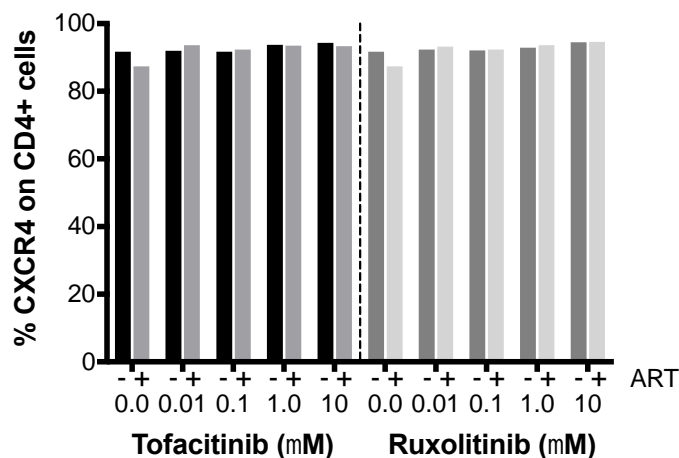**B****CXCR4+(Mean)**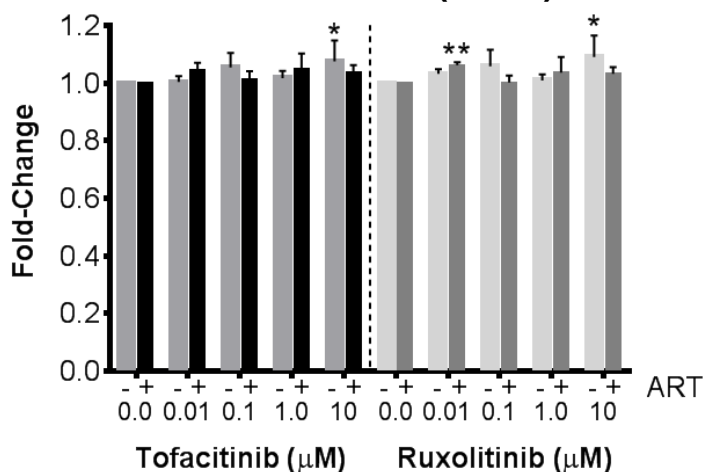

Supplement: S6 Fig — HIV coreceptor CXCR4 was quantified in CD4+ T cells isolated from viremic donors and cultured for 6 days as in (Fig 2A and 2B). Percentage of CD4 cells expressing CXCR4 from individual donors (A). To account for inter-patient variability in baseline values, results in B are reported as the fold change versus DMSO controls. 0.0 μM represents the average of all assays completed using % DMSO equivalent to Jak inhibitor concentrations. Error bars represent S.E.M. and statistical significance determined by two-way ANOVA followed by Sidak’s multiple comparison post-test: *p < 0.05, **p < 0.01, ***p < 0.001 and ****p < 0.0001. (PDF) [file ppat.1006740.s006.pdf]

**A****p24 Fold-Change**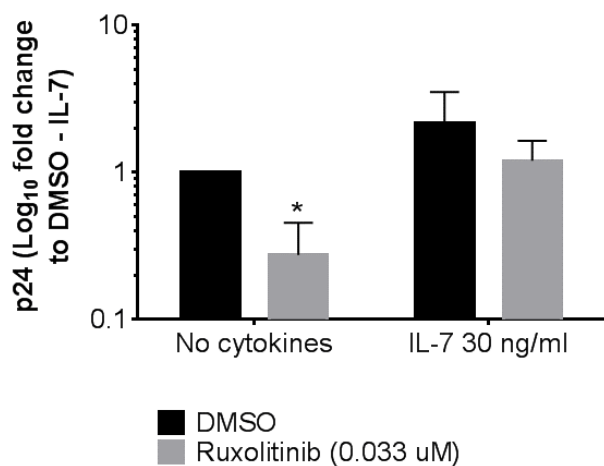**B****p24 (pg/ml) MHCR011b**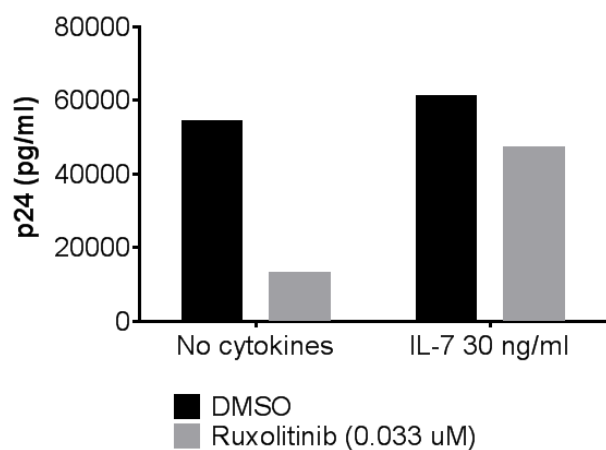**p24 (pg/ml) MHCR003b**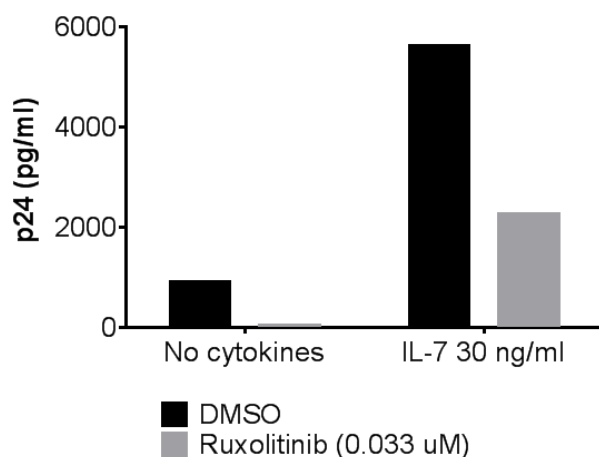**p24 (pg/ml) MA003**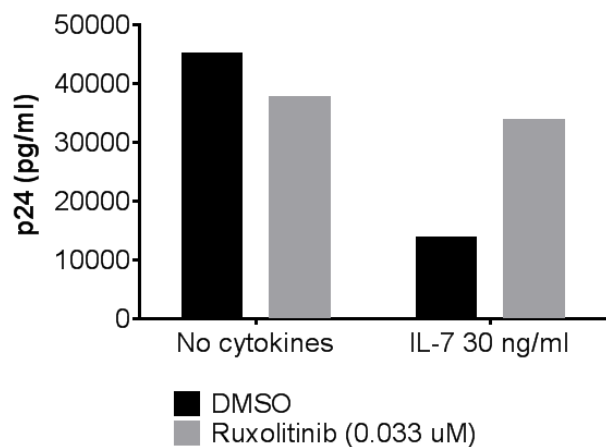**p24 (pg/ml) MA004**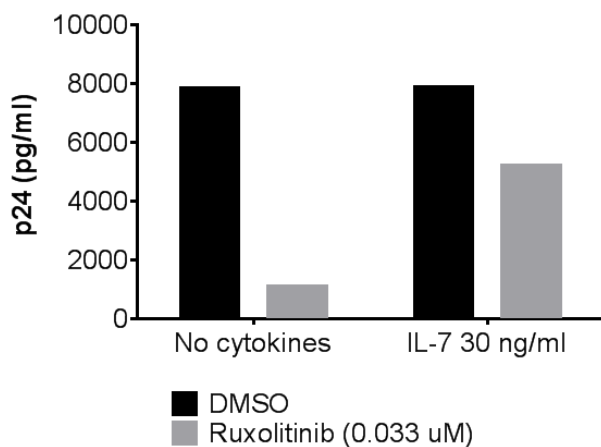

Supplement: S7 Fig — CD4 T cells from viremic donors (n = 4) were pre-incubated with anti-CD3/CD28 and 33 nM Ruxolitinib 30 min prior to addition of IL-7 (30 ng/mL). p24 was measured after 6 days in culture. Error bars represent S.E.M. and statistical significance determined by paired T-test (A), where DMSO controls without cytokine versus DMSO control + IL-7 was compared (paired t-test) and Ruxolitinib (no cytokine) was compared to ruxolitinib (+ IL-7) (paired t-test). * p < 0.05 compared to no cytokine addition. p24 measurements from each individual donor (B). (PDF) [file ppat.1006740.s007.pdf]

**A****Proliferation**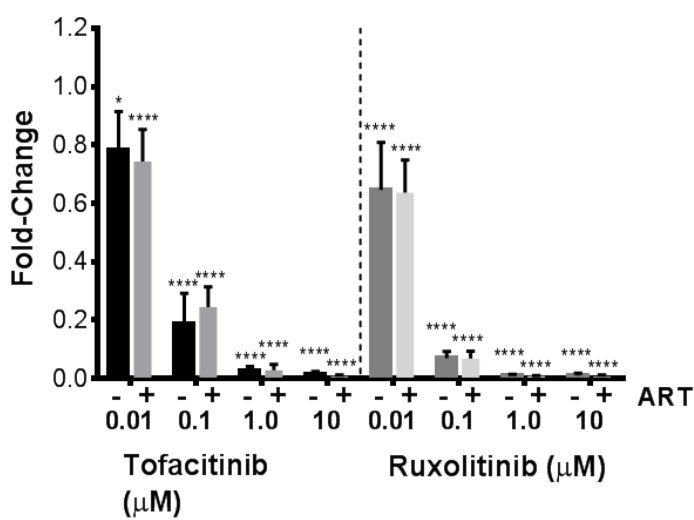**B****CD25+**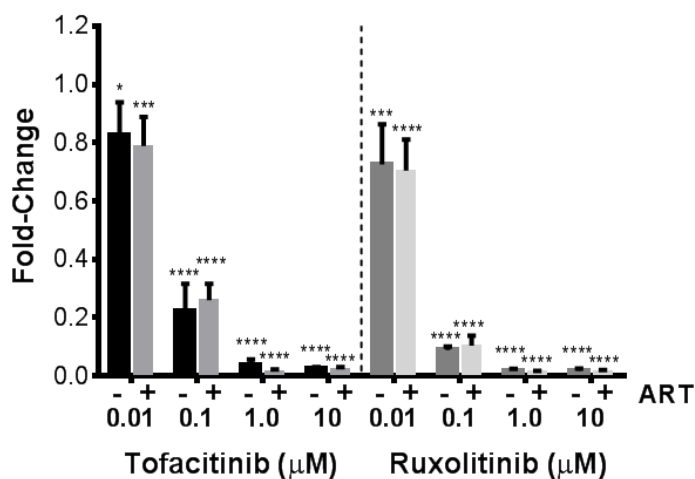**C****CD38/HLADR+**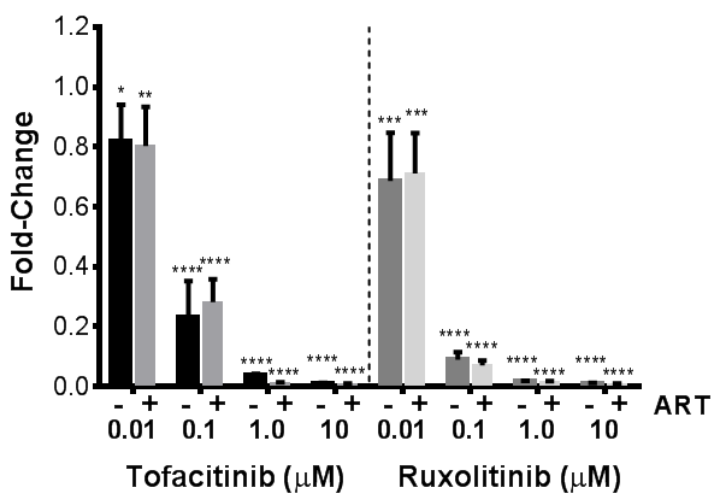**D****PD1+**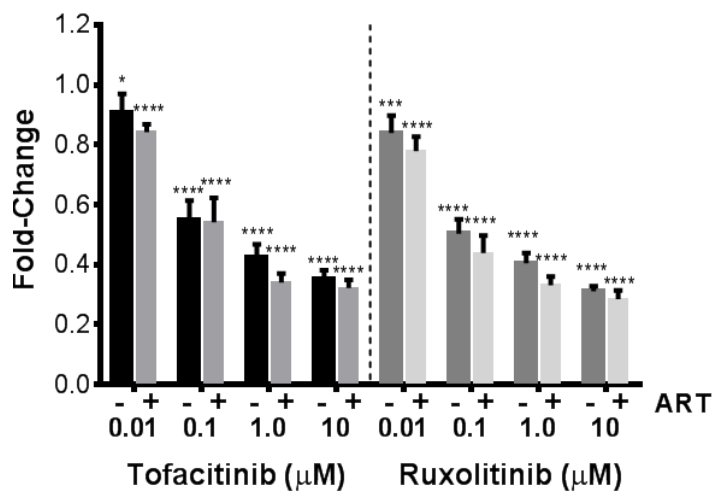

Supplement: S8 Fig — Cell proliferation (A) and activation (B-D) as measured by flow cytometry in enriched CD4+ T cells isolated from viremic donors and cultured for 6 days with CD3/28 and increasing concentrations of Jak inhibitors in the absence of antiretroviral agents [(-); designed to observe the effect of ruxolitinib alone, in the presence of ongoing replication] or presence of 180 nM zidovudine, 100 nM efavirenz, 200 nM raltegravir [(+); to observe the effect of ruxolitinib when all spreading infection is inhibited] (n = 5). Percentage of cells expressing CD25 (B), HLA-DR/CD38 (C), PD-1 (D) and low levels of Cell Trace Violet [CTV] (A). To account for inter-patient variability in baseline values, results are reported as the fold change versus DMSO treated control cells. Activation and proliferation markers by the latter are normalized to 1. Error bars represent S.E.M. and statistical significance determined by two-way ANOVA followed by Sidak’s multiple comparison post-test: * p < 0.05, ** p < 0.01, *** p < 0.001 and **** p < 0.0001. (PDF) [file ppat.1006740.s008.pdf]

CD25+ (MCHR008)

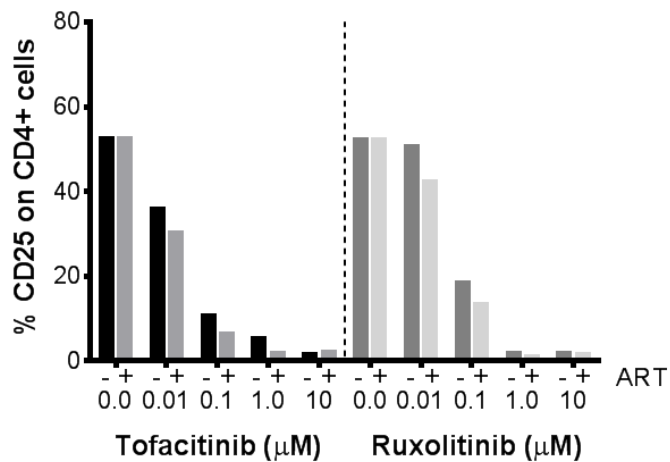

CD25+ (MCHR009)

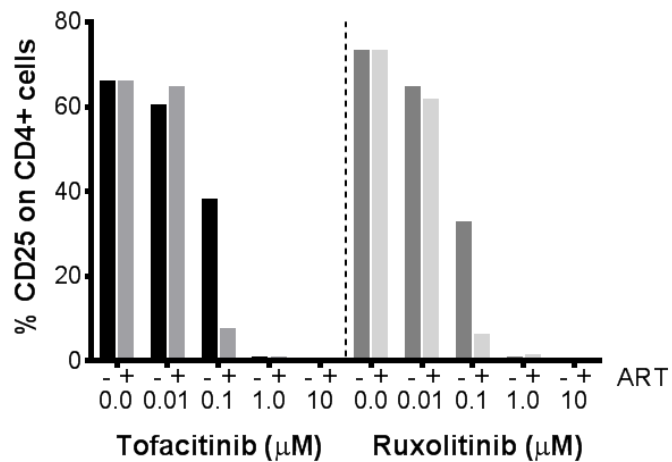

CD25+ (MCHR010)

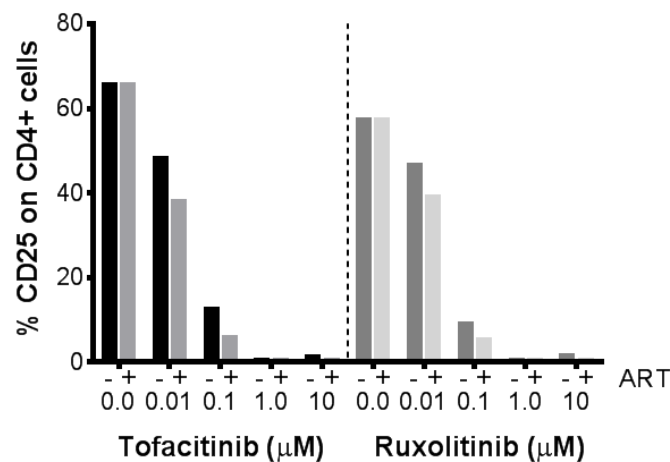

CD25+ (MCHR007b)

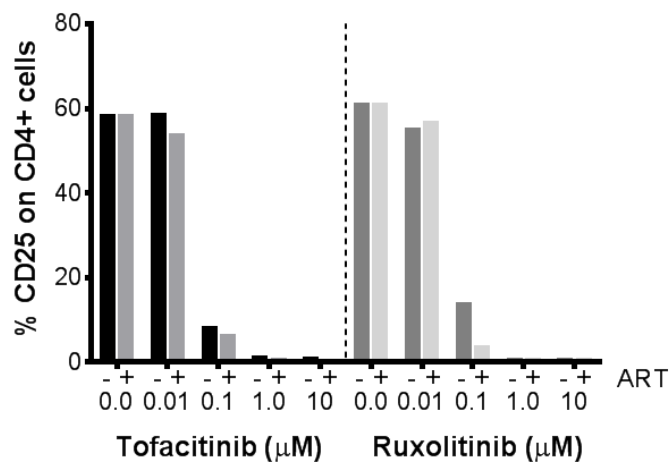

CD25+ (MCHR011)

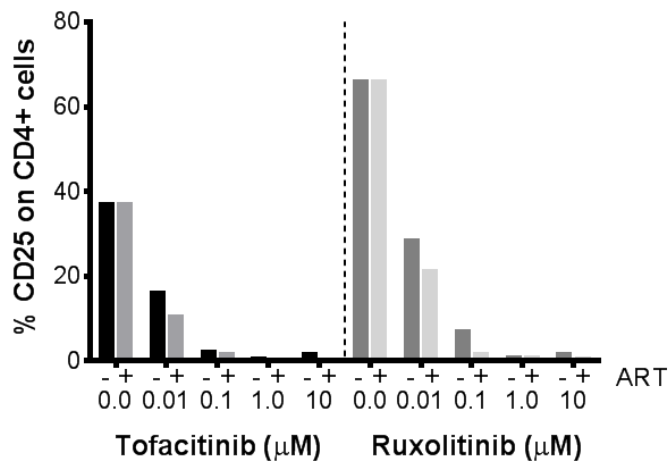

Supplement: S10 Fig — CD25 expression as measured in S8 Fig in individual donors. (PDF) [file ppat.1006740.s010.pdf]

**CD38/HLADR+ (MCHR008)**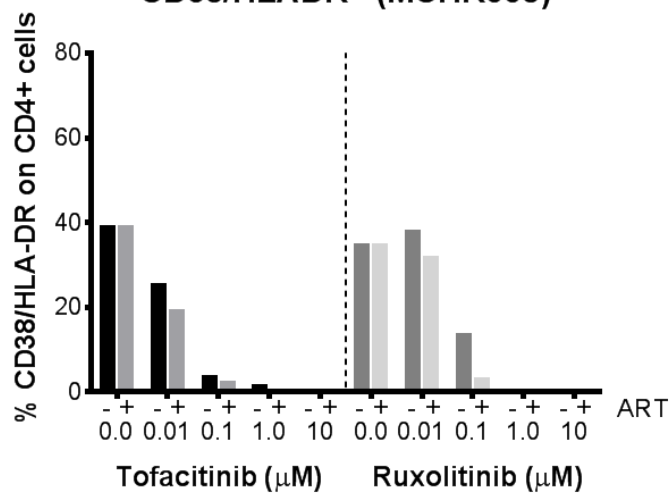**CD38/HLADR+ (MCHR009)**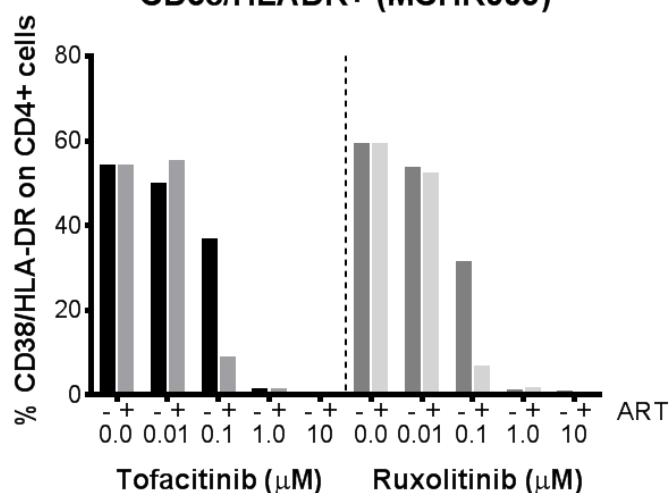**CD38/HLADR+ (MCHR010)**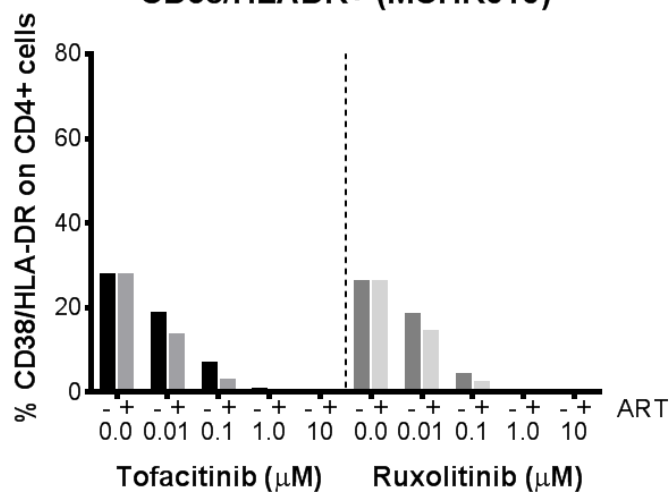**CD38/HLADR+ (MCHR007b)**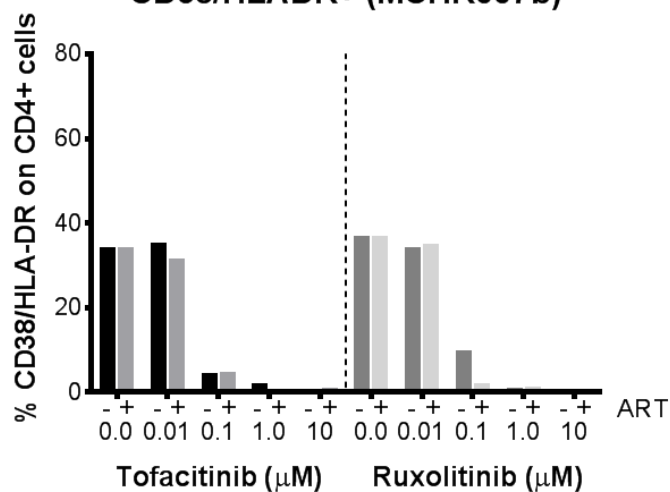**CD38/HLADR+ (MCHR011)**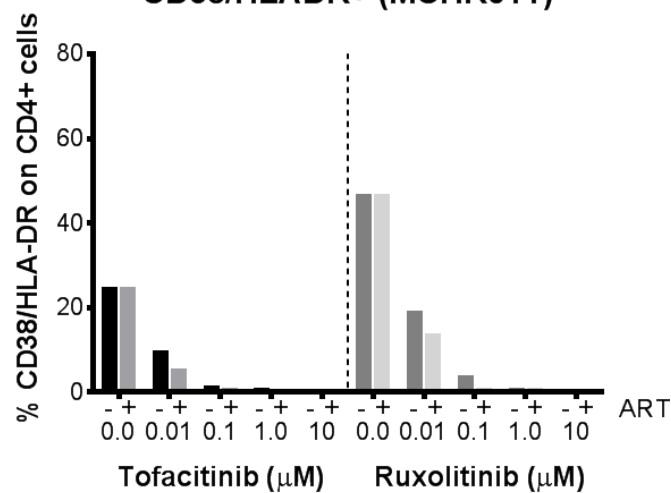

Supplement: S11 Fig — CD38/HLA-DR expression as measured in S8 Fig in individual donors. (PDF) [file ppat.1006740.s011.pdf]

**PD1+ (MCHR008)**

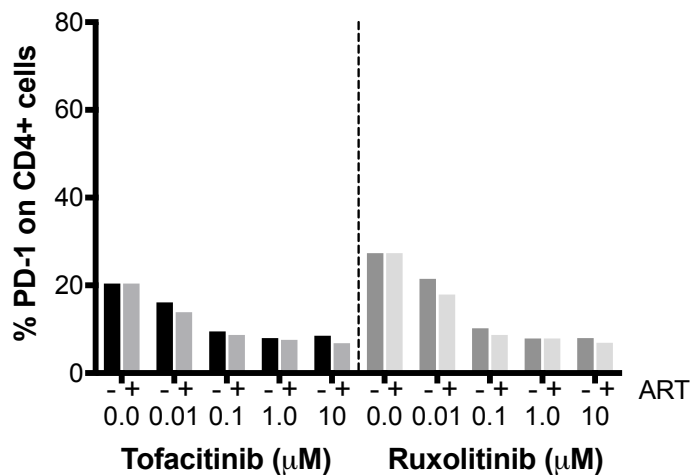

**PD1+ (MCHR009)**

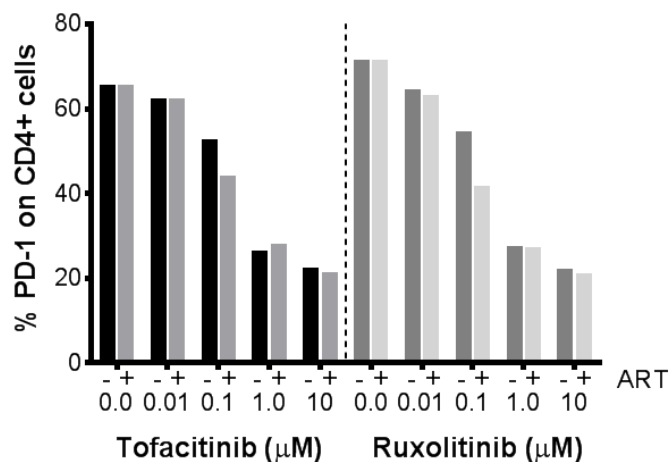

**PD1+ (MCHR010)**

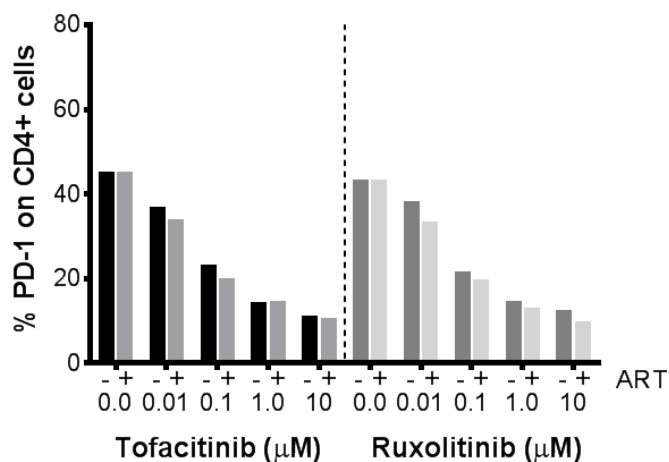

**PD1+ (MCHR007b)**

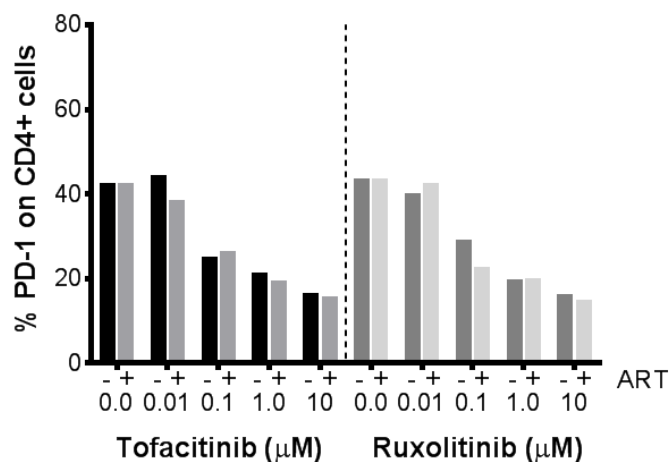

**PD1+ (MCHR011)**

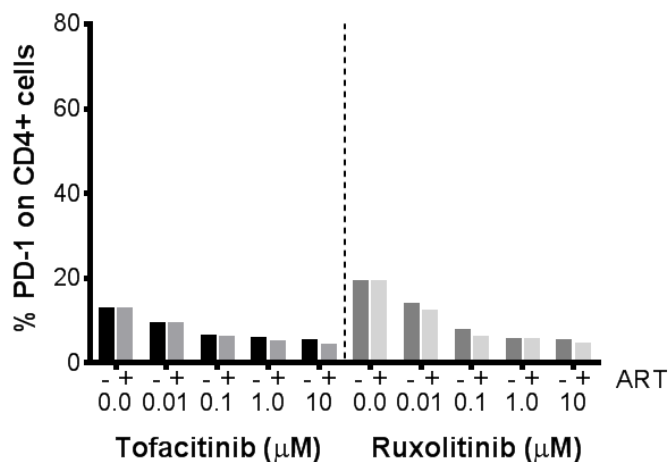

Supplement: S12 Fig — PD-1 expression as measured in S8 Fig in individual donors. (PDF) [file ppat.1006740.s012.pdf]

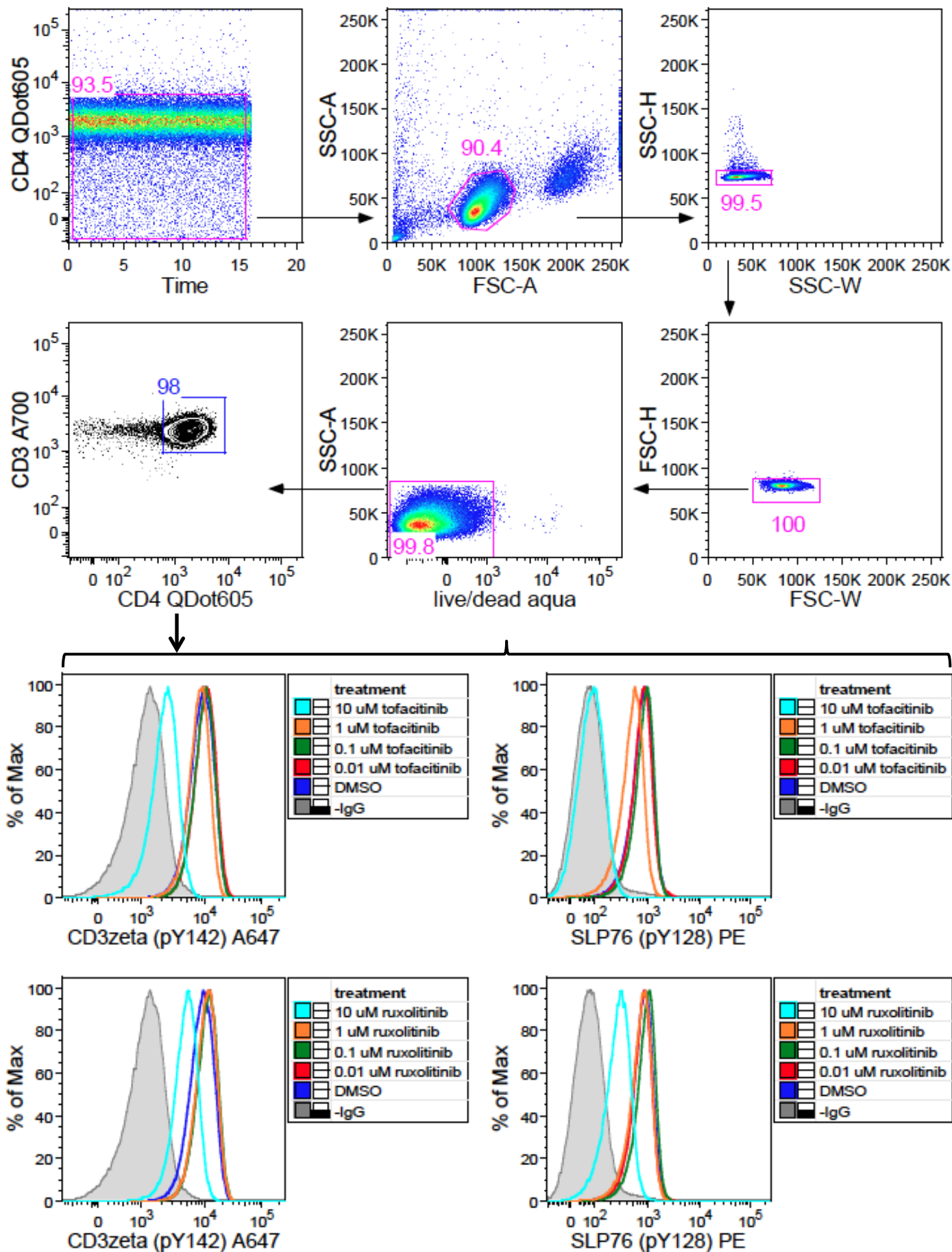

**S13 Fig**

Supplement: S13 Fig — (PDF) [file ppat.1006740.s013.pdf]

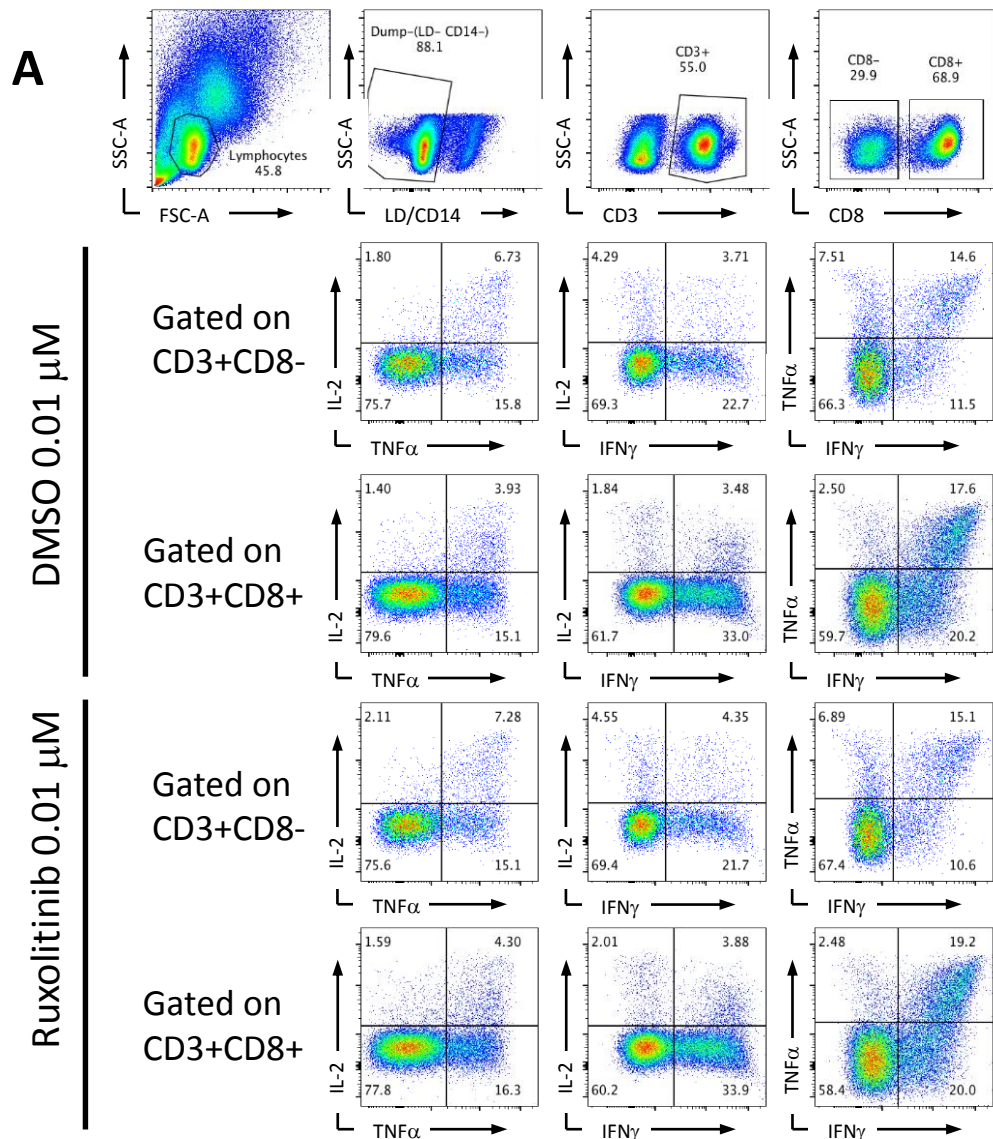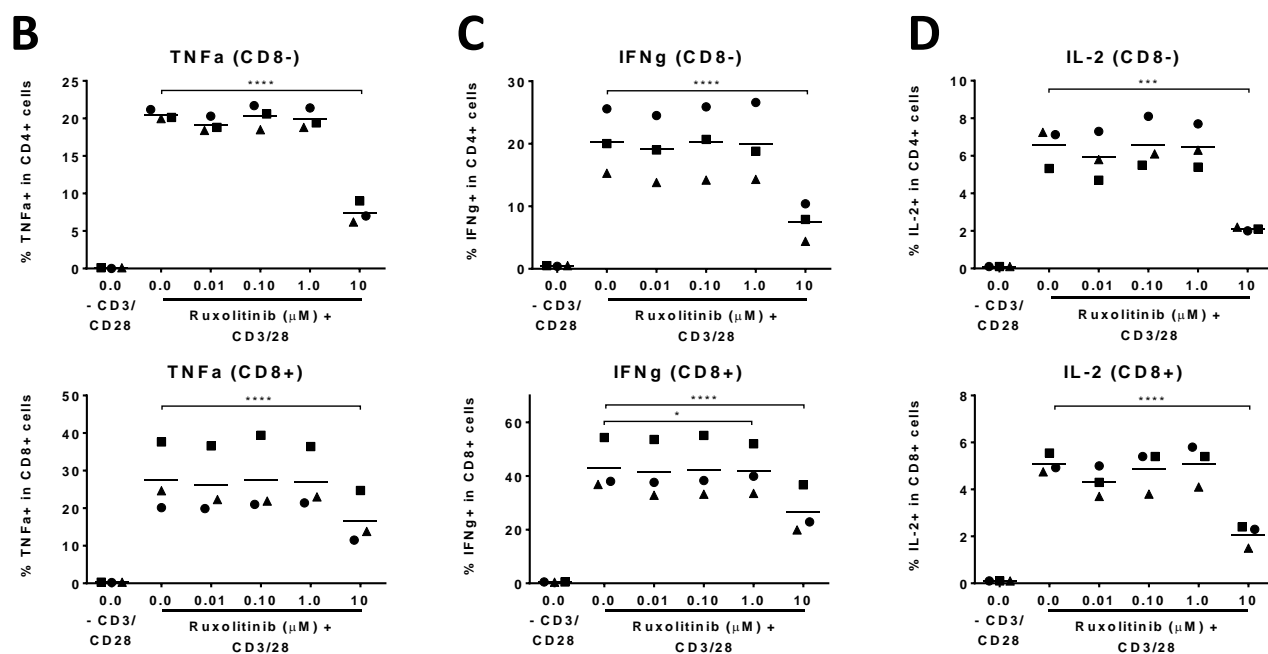

Supplement: S14 Fig — Gating strategy (A) and impact of ruxolitinib on (B) TNF-α+, (C) IFN-γ+ or (D) IL-2+ expressing CD4 or CD8 cells. Mean cytokine production (% of IL-2+, TNF-α+ or IFN-γ+ positive cells) in CD3+CD8- cells or CD3+CD8+ cells as measured by flow cytometry in PBMC isolated from HIV negative donors and stimulated for 6 hr with aCD3/CD28, Brefeldin A (5 μg/ml) and increasing concentrations of Ruxoltinib versus DMSO treated cells (n = 3). 0.0 μM represents the average of all assays completed using % DMSO equivalent to Jak inhibitor concentrations. Statistical significance determined by two-way ANOVA followed by Sidak’s multiple comparison post-test: *p < 0.05, **p < 0.01, ***p < 0.001 and ****p < 0.0001. (PDF) [file ppat.1006740.s014.pdf]

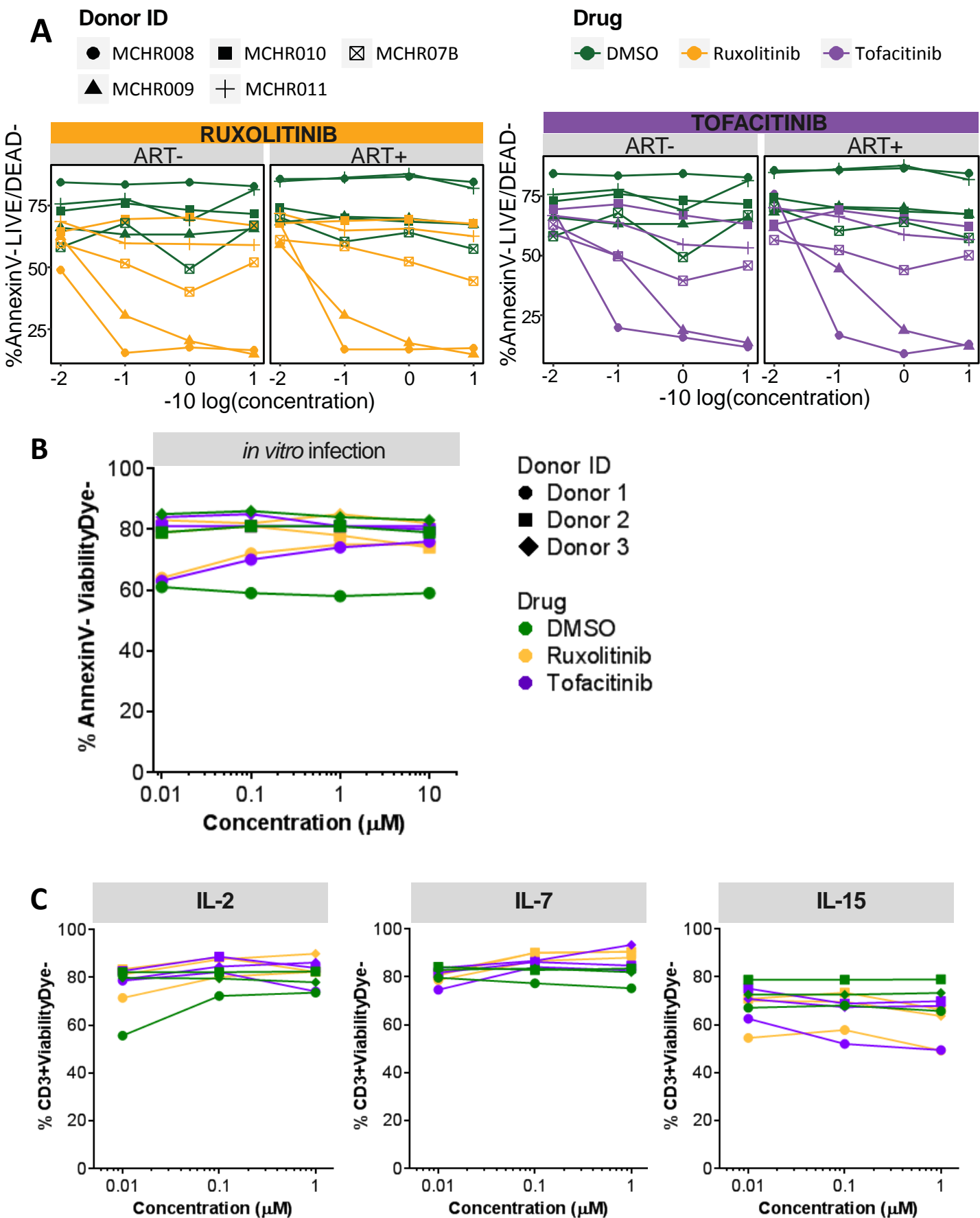

**S15 Fig**

Supplement: S15 Fig — The frequency of live cells (AnnexinV- and Live/Dead viability mearker -) as measured by flow cytometry in enriched CD4+ T cells isolated from viremic donors and cultured for 6 days with CD3/28 and increasing concentrations of Jak inhibitors in the absence of antiretroviral agents [(ART-); designed to observe the effect of ruxolitinib or tofacitinib alone, in the presence of ongoing replication] or presence of 180 nM zidovudine, 100 nM efavirenz, 200 nM raltegravir [(ART+); to observe the effect of ruxolitinib or tofacitinib when all spreading infection is inhibited] (n = 5) (A). The frequency of live cells (AnnexinV- and Live/Dead viability marker -) measured after 3 day culture of in vitro infection of CD4+ cells from 3 healthy donros in the presence of increasing concentrations of Jak inhibitors with ART as described in Fig 2D (B). Frequency of live cells (Live/Dead viability marker -) measured after 6 day culture of CD4+ cells from healthy donors in the presence of increasing concentrations of Jak inhibitors and IL-2, -7 or -15 as described in Fig 1C. (PDF) [file ppat.1006740.s015.pdf]

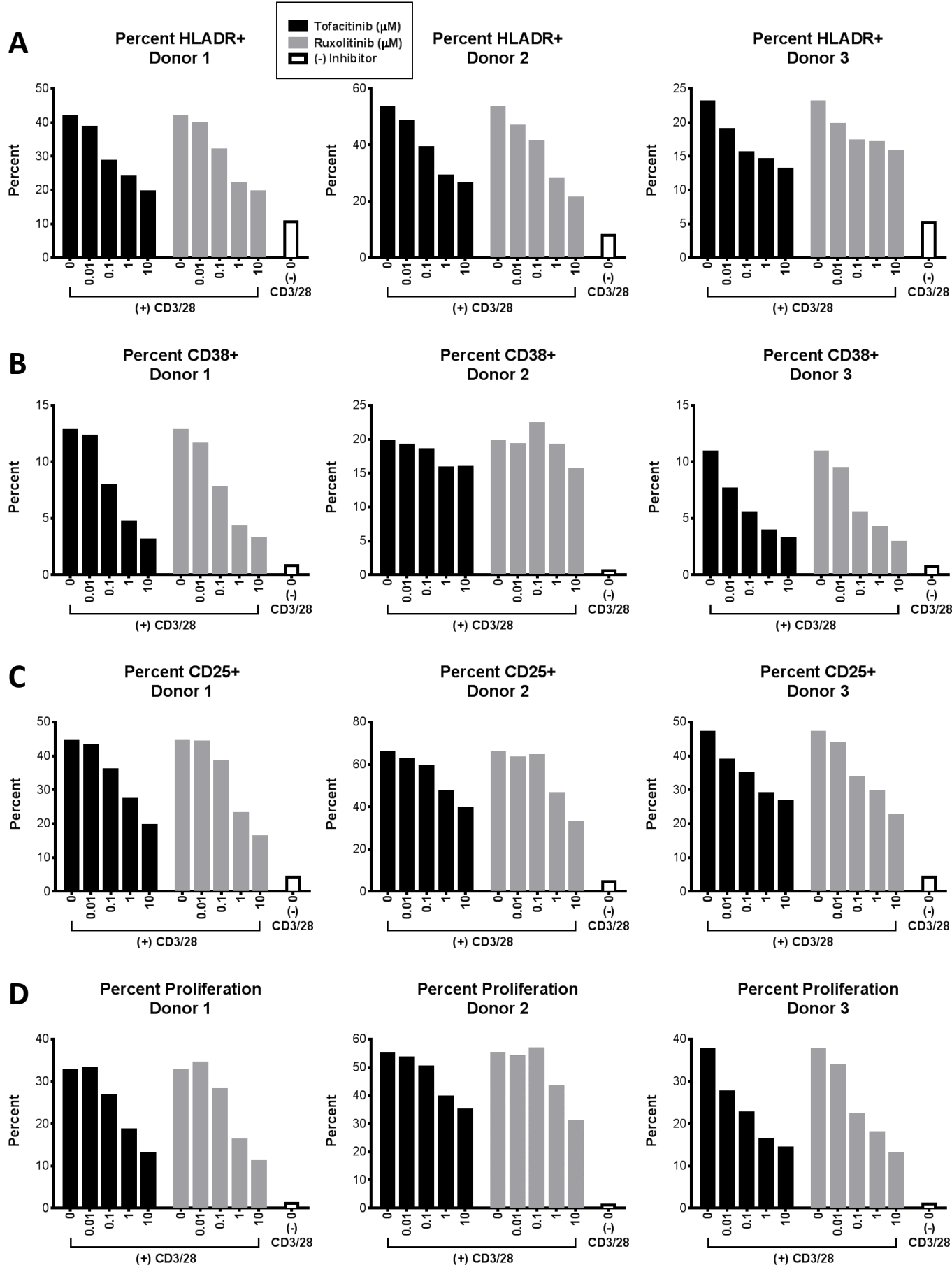

**S16 Fig**

Supplement: S16 Fig — Activation (A-C) and proliferation (D) measured by flow cytometry of in vitro infected CD4+ T cells after 3 days culture with anti-CD3/28 in the presence of increasing concentrations of Jak inhibitors and ART (180 nM zidovudine, 100 nM efavirenz, 200 nM raltegravir) [N = 3] as described in Fig 2D. Percentage of cells expressing HLA-DR (A), CD38 (B), CD25 (C) and low levels of Cell Trace Violet [CTV] (D). (PDF) [file ppat.1006740.s016.pdf]

log10(Mean Fluorescence Intensity)

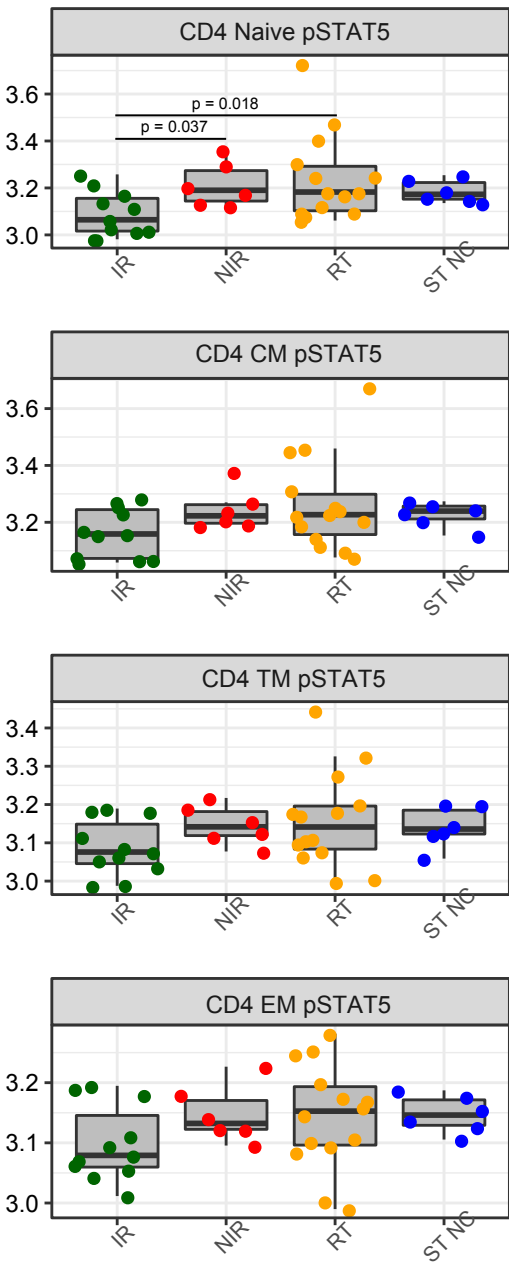

**S17 Fig**

Donor Class

Supplement: S17 Fig — pSTAT5 MFI between non immune responders (NIR), Immune Responders (IR), Successfully Treated Non Classified (ST NC) and Recently Treated (RT) was determined in Naïve, CM, TM and EM CD4 T cell subsets by the Wiloxon rank test. A p-value < 0.05 was considered statistically significant. (PDF) [file ppat.1006740.s017.pdf]

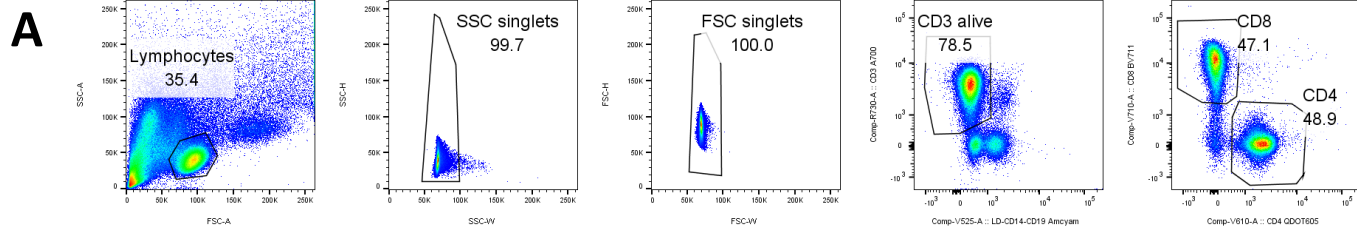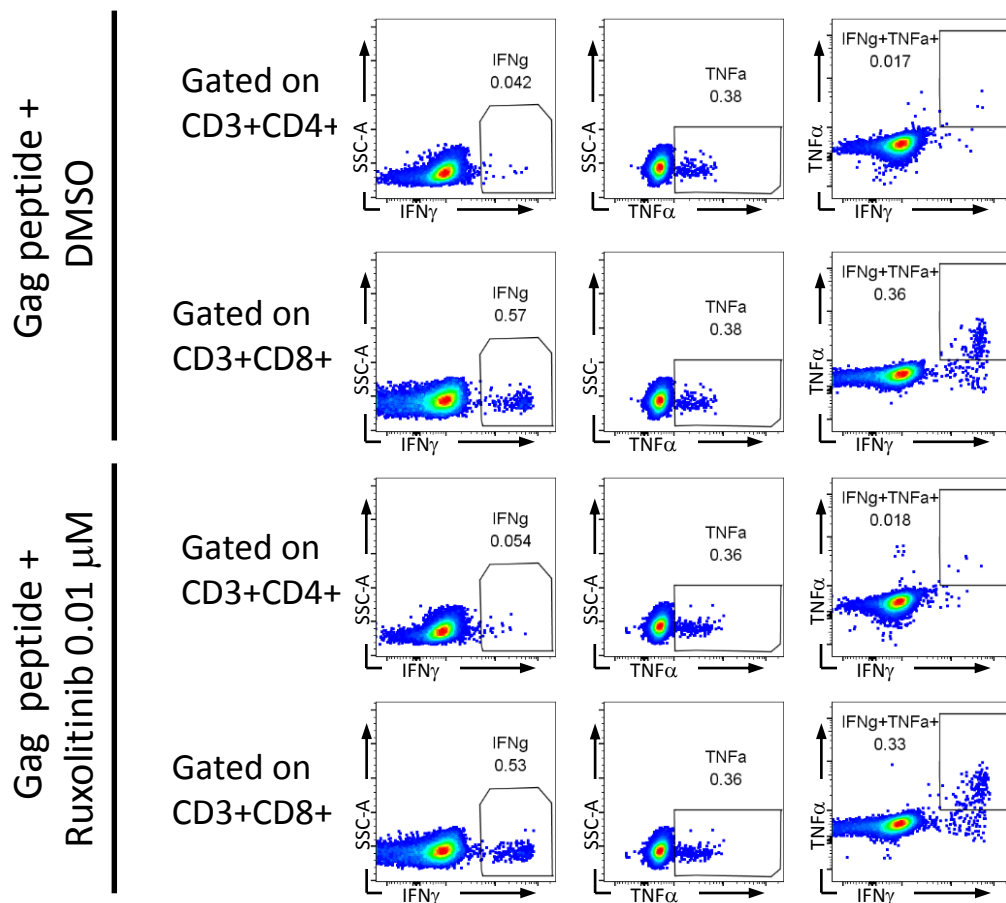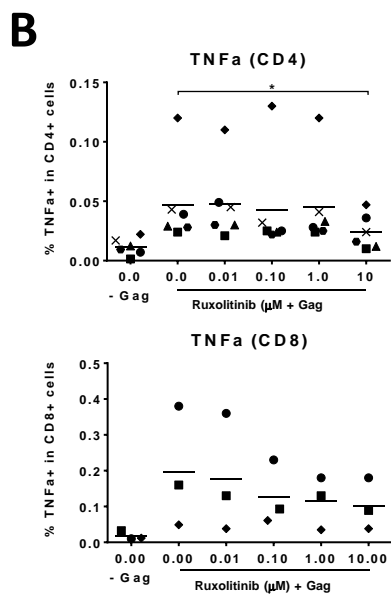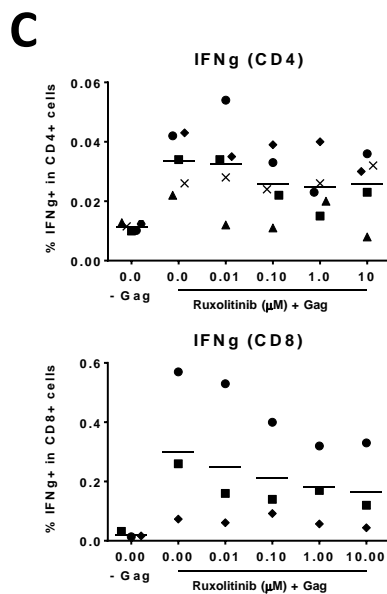

Supplement: S18 Fig — Gating strategy (A) and impact of ruxolitinib on (B) TNF-α+ or (C) IFN-γ+ expressing CD4 or CD8 cells. Mean percent TNF-α+ (n = 6) or IFN-γ+ (n = 5) cells in CD3+CD4+ cells or CD3+CD8+ cells (n = 3) as measured by flow cytometry in PBMC isolated from stably treated, HIV positive donors and stimulated for 6 hr with 1 μg/ml gag-peptide, Brefeldin A (5 μg/ml) and increasing concentrations of Ruxoltinib versus DMSO treated cells. 0.0 μM represents the average of all assays completed using % DMSO equivalent to Jak inhibitor concentrations. Statistical significance determined by paired Wilcoxon rank sum test. (PDF) [file ppat.1006740.s018.pdf]
