# Supplementary material for: Novel mechanisms to inhibit HIV reservoir seeding using Jak inhibitors
Source: PLoS Pathog. 2017 Dec 21;13(12):e1006740. doi: 10.1371/journal.ppat.1006740 (PMC5739511; doi:10.1371/journal.ppat.1006740)
Supplement: S2 Fig — STAT1, STAT3 or STAT5 phosphorylation (% in CD4+ T cells and CD14+ monocytes) was measured by flow cytometry in PBMC isolated from HIV negative donors and stimulated for 15 min with 10,000 Units IFN-α (A-D) or 10 ng/ml IL-10 (E-F) (n = 3) and increasing concentrations (0.01, 0.1, and 1.0 μM) of ruxolitinib. 0.0 μM represents the average of all assays completed using % DMSO equivalent to Jak inhibitor concentrations. Error bars represent standard deviation and statistical significance determined by two-way ANOVA followed by Sidak’s multiple comparison post-test: *p < 0.05, **p < 0.01, ***p < 0.001 and ****p < 0.0001. (PDF) [file ppat.1006740.s002.pdf]

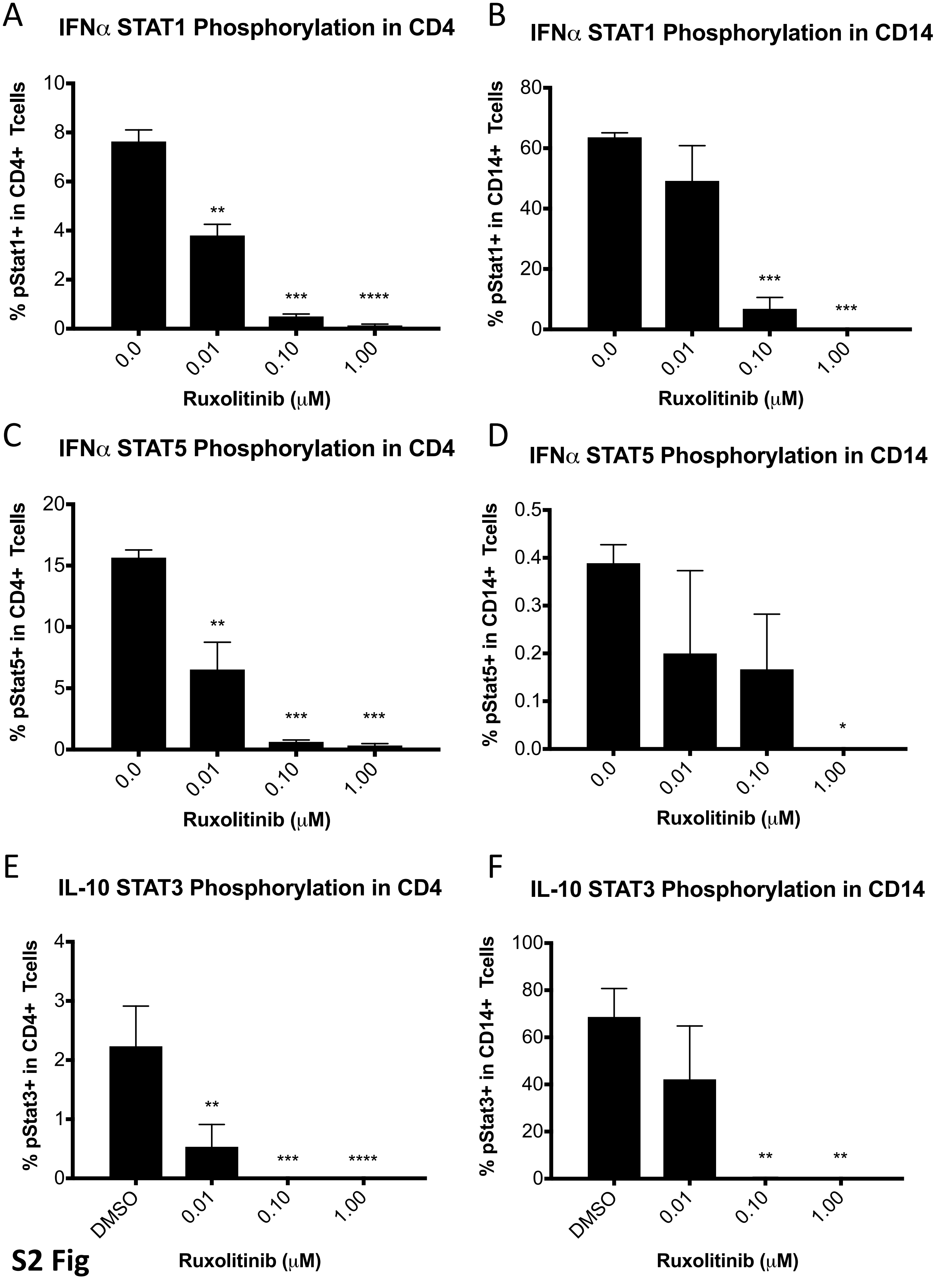
A
IFNα STAT1 Phosphorylation in CD4
% pStat1+ in CD4+ Tcells
10
8
6
4
2
0
**
***
****
0.0
0.01
0.10
1.00
Ruxolitinib (μM)
B
IFNα STAT1 Phosphorylation in CD14
% pStat1+ in CD14+ Tcells
80
60
40
20
0
***
***
0.0
0.01
0.10
1.00
Ruxolitinib (μM)
C
IFNα STAT5 Phosphorylation in CD4
% pStat5+ in CD4+ Tcells
20
15
10
5
0
**
***
***
0.0
0.01
0.10
1.00
Ruxolitinib (μM)
D
IFNα STAT5 Phosphorylation in CD14
% pStat5+ in CD14+ Tcells
0.5
0.4
0.3
0.2
0.1
0.0
*
0.0
0.01
0.10
1.00
Ruxolitinib (μM)
E
IL-10 STAT3 Phosphorylation in CD4
% pStat3+ in CD4+ Tcells
4
3
2
1
0
**
***
****
DMSO
0.01
0.10
1.00
Ruxolitinib (μM)
F
IL-10 STAT3 Phosphorylation in CD14
% pStat3+ in CD14+ Tcells
100
80
60
40
20
0
**
**
DMSO
0.01
0.10
1.00
Ruxolitinib (μM)

S2 Fig
