# Supplementary material for: Novel mechanisms to inhibit HIV reservoir seeding using Jak inhibitors
Source: PLoS Pathog. 2017 Dec 21;13(12):e1006740. doi: 10.1371/journal.ppat.1006740 (PMC5739511; doi:10.1371/journal.ppat.1006740)
Supplement: S3 Fig — Raw data of viral replication measured by ELISA p24 in cell-free supernatants of enriched CD4+ T cells isolated from 5 viremic donors and stimulated for 6 days with anti-CD3/28 in the presence of increasing concentrations of Jak inhibitors without ART. (PDF) [file ppat.1006740.s003.pdf]

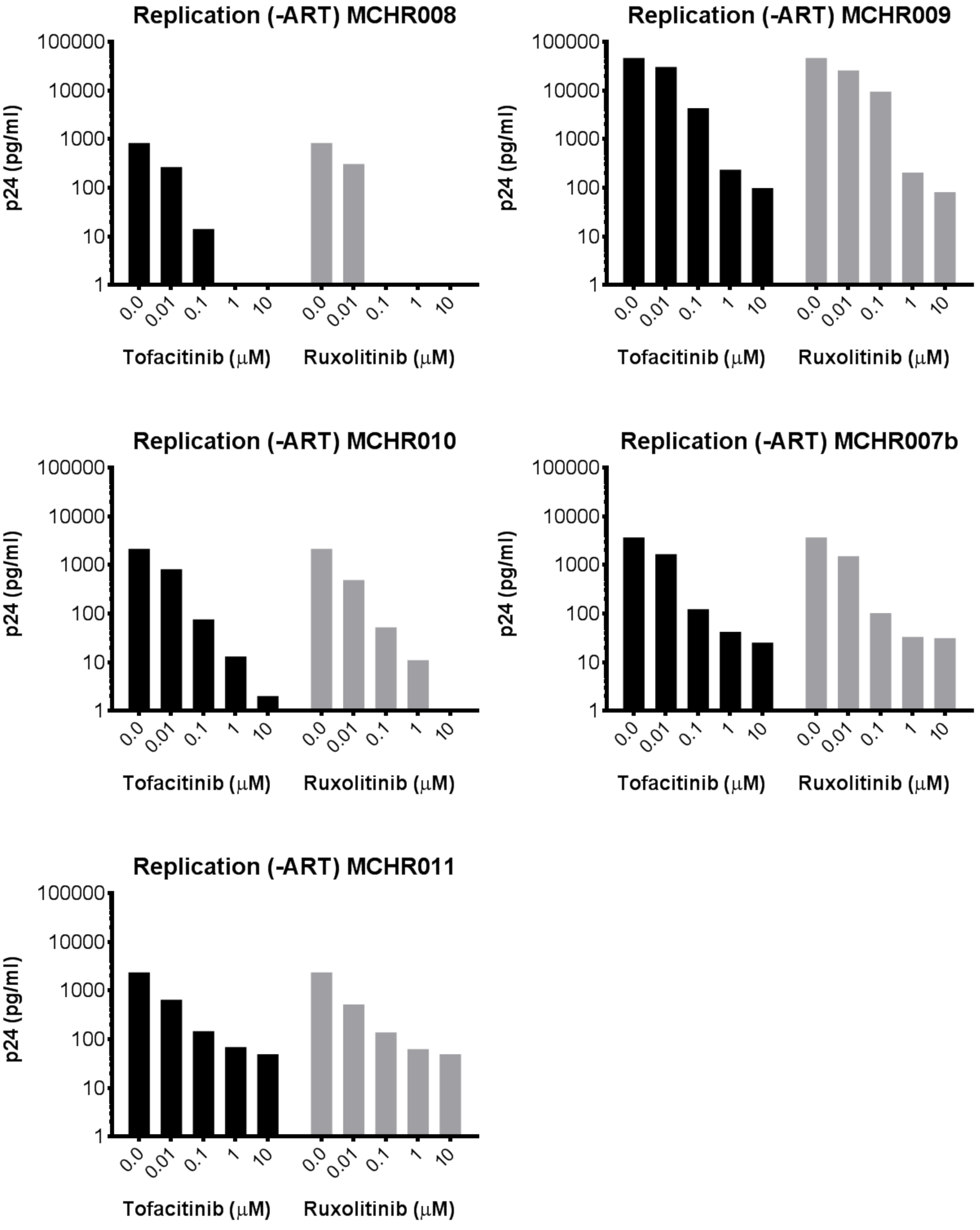
Replication (-ART) MCHR008
Replication (-ART) MCHR009
Replication (-ART) MCHR010
Replication (-ART) MCHR007b
Replication (-ART) MCHR011
p24 (pg/ml)
100000
10000
1000
100
10
1
0.0
0.01
0.1
1
10
Tofacitinib (μM)
Ruxolitinib (μM)

S3 Fig
