# Supplementary material for: Novel mechanisms to inhibit HIV reservoir seeding using Jak inhibitors
Source: PLoS Pathog. 2017 Dec 21;13(12):e1006740. doi: 10.1371/journal.ppat.1006740 (PMC5739511; doi:10.1371/journal.ppat.1006740)
Supplement: S9 Fig — Cell proliferation as measured in S8 Fig in individual donors. (PDF) [file ppat.1006740.s009.pdf]

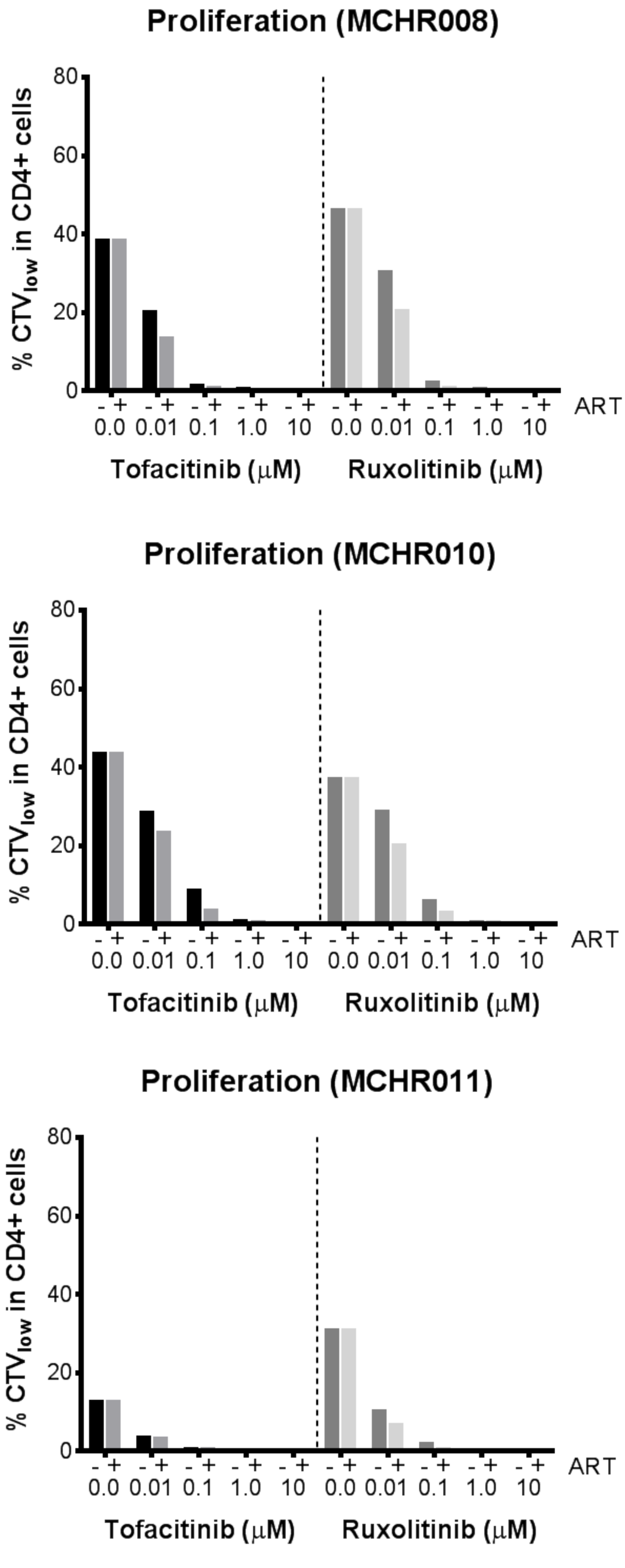
Proliferation (MCHR008)
Proliferation (MCHR009)
Proliferation (MCHR010)
Proliferation (MCHR007b)
Proliferation (MCHR011)
% CTV$_{low}$ in CD4+ cells
0
20
40
60
80
ART
0.0 0.01 0.1 1.0 10
Tofacitinib (μM)
Ruxolitinib (μM)

S9 Fig
