# Supplementary material for: Novel mechanisms to inhibit HIV reservoir seeding using Jak inhibitors
Source: PLoS Pathog. 2017 Dec 21;13(12):e1006740. doi: 10.1371/journal.ppat.1006740 (PMC5739511; doi:10.1371/journal.ppat.1006740)
Supplement: S1 Table — (PDF) [file ppat.1006740.s019.pdf]

S1 Table. Profile of ART-treated HIV infected subjects including CD4, CD8 T cell counts, viral load, time individual has been HIV-infected, and whether individual is receiving ART.

Patient Code	Class (VL)	Class (VL and CD4 count)	nadir CD4	pre-ART HIV RNA	CD4	CD8	CD4/ CD8	VLc	ART	T-inf	Aviremic
IDR 236	aviremic	IR	323	N/A	916	1307	0.70	98	Yes	16	2
IDR 237	aviremic	IR	249	50034	618	579	1.07	< 50	Yes	7	4
IDR 239	aviremic	IR	276	8691	687	1005	0.68	< 50	Yes	15	7
IDR 244	aviremic	IR	360	217	616	1230	0.50	< 50	Yes	25	8
ST 016	aviremic	IR	N/A	N/A	558	972	0.57	< 50	Yes	25	10
IDR 266	aviremic	IR	207	15959	520	560	0.93	< 50	Yes	5	4
IDR 271	aviremic	IR	255	408430	653	1158	0.56	< 50	Yes	10	9
IDR 276	aviremic	IR	170	N/A	419	356	1.18	< 50	Yes	6	5
IDR 248	aviremic	IR	10	68628	517	718	0.72	< 50	Yes	6	5
IDR 279	aviremic	IR	244	8189	1139	1034	1.10	< 50	Yes	24	2
IDR 283	aviremic	IR	219	7395	649	450	1.44	< 50	Yes	6	5
IDR 267	aviremic	NIR	133	4781	372	631	0.59	< 50	Yes	6	5
IDR 273	aviremic	NIR	69	6728	115	467	0.25	< 50	Yes	3	2
IDR 256	aviremic	NIR	3	52953	97	410	0.24	< 50	Yes	24	3
IDR 258	aviremic	NIR	54	32864	288	667	0.43	< 50	Yes	23	3
IDR 259	aviremic	NIR	6	62174	326	677	0.48	< 50	Yes	19	4
IDR 281	aviremic	NIR	331	111	340	262	1.30	< 50	Yes	23	4
IDR 238	aviremic	RT	63	7161	63	346	0.18	< 50	Yes	2	1
# 888	aviremic	RT	N/A	N/A	797	934	0.85	< 50	Yes	2	1
IDR 241	aviremic	RT	252	N/A	469	969	0.48	< 50	Yes	11	3
IDR 240	aviremic	RT	168	50721	384	682	0.56	< 50	Yes	2	1
IDR 261	aviremic	RT	301	34117	537	637	0.84	< 50	Yes	2	1
IDR 262	aviremic	RT	251	24905	502	747	0.67	< 50	Yes	2	1
IDR 263	aviremic	RT	279	12996	455	491	0.93	< 50	Yes	7	1
IDR 264	aviremic	RT	66	N/A	112	657	0.17	< 50	Yes	8	1

IDR 246	aviremic	RT	9	N/A	447	348	1.28	54	Yes	17	2
IDR 247	aviremic	RT	326	34363	488	514	0.95	< 50	Yes	14	1
IDR 277	aviremic	RT	162	19537	275	1146	0.24	< 50	Yes	1	1
IDR 278	aviremic	RT	1	318432	260	533	0.49	< 50	Yes	15	1
IDR 280	aviremic	RT	50	N/A	209	837	0.25	< 50	Yes	2	1
IDR 245	aviremic	RT	42	162094	210	686	0.31	< 50	Yes	4	2
IDR 265	aviremic	ST NC	132	N/A	400	738	0.54	< 50	Yes	5	4
IDR 275	aviremic	ST NC	167	20753	445	873	0.51	< 50	Yes	7	3
IDR 257	aviremic	ST NC	204	N/A	369	350	1.05	< 50	Yes	5	4
IDR 260	aviremic	ST NC	140	66697	382	229	1.67	< 50	Yes	5	4
IDR 282	aviremic	ST NC	28	32100	449	582	0.77	< 50	Yes	20	4
IDR 284	aviremic	ST NC	114	263415	569	979	0.58	< 50	Yes	6	5

Detailed Class: IR (VL<50, CD4 count > 500 for 2 years), NIR (VL<50, CD4 count < 350 for 2 years), ST NC (VL<50, CD4 count in between 350 and 500), RT (VL<50 for 1 year or less)
nadir CD4: Cells per ml
pre-ART HIV RNA: copies/ml
CD4 and CD8: Cells per ml
VLc: Viral load (copies/ml)
ART: Antiretroviral Therapy; None: never received; Yes: received
T-inf: Time since HIV infection (Year)
Aviremic: Time of undetectable viremia (with or without ART) in years
IR: Immune Responder
NIR: Non Immune Responder
ST NC: Successfully treated Non Classified (CD4 T cell counts in between IR and NIR)
RT: Recently Treated
